# Biochemical and Structural Characterization of an Unusual and Naturally Split Class 3 Intein

**DOI:** 10.1002/cbic.202000509

**Published:** 2020-09-30

**Authors:** Simon Hoffmann, Tobias M. E. Terhorst, Rohit K. Singh, Daniel Kümmel, Shmuel Pietrokovski, Henning D. Mootz

**Affiliations:** ^1^ Institute of Biochemistry University of Muenster Corrensstraße 36 48149 Münster Germany; ^2^ Department of Molecular Genetics Weizmann Institute of Science Rehovot 76100 Israel

**Keywords:** crystal structures, post-translational modifications, protein mechanisms, protein splicing, split inteins

## Abstract

Split inteins are indispensable tools for protein engineering because their ligation and cleavage reactions enable unique modifications of the polypeptide backbone. Three different classes of inteins have been identified according to the nature of the covalent intermediates resulting from the acyl rearrangements in the multistep protein‐splicing pathway. Class 3 inteins employ a characteristic internal cysteine for a branched thioester intermediate. A bioinformatic database search of non‐redundant protein sequences revealed the absence of split variants in 1701 class 3 inteins. We have discovered the first reported split class 3 intein in a metagenomics data set and report its biochemical, mechanistic and structural analysis. The AceL NrdHF intein exhibits low sequence conservation with other inteins and marked deviations in residues at conserved key positions, including a variation of the typical class‐3 WCT triplet motif. Nevertheless, functional analysis confirmed the class 3 mechanism of the intein and revealed excellent splicing yields within a few minutes over a wide range of conditions and with barely detectable cleavage side reactions. A high‐resolution crystal structure of the AceL NrdHF precursor and a mutagenesis study explained the importance and roles of several residues at the key positions. Tolerated substitutions in the flanking extein residues and a high affinity between the split intein fragments further underline the intein's future potential as a ligation tool.

## Graphical abstract

**Split happens**: Analysis of metagenomics data led to the identification of the first class 3 split intein. Bioinformatics studies, biochemical characterization, protein crystallography and mutational analysis revealed a high affinity of the intein fragments, mechanistic roles of key residues and identified the AceL NrdHF intein as a promising intein for biotechnological applications.

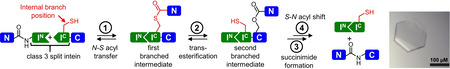

## Introduction

Inteins (intervening proteins) are protein domains inserted into host proteins that are found in all domains of life. They remove themselves out of their precursor proteins in an autocatalytic process called protein splicing, in which the flanking N‐ and C‐terminal exteins are ligated to form the mature host protein.^[1]^ In protein *trans*‐splicing the intein domain is split into an N‐ and a C‐terminal fragment (Int^N^ and Int^C^) and hence the extein sequences are ligated from two separate precursor proteins.^[2]^ Both regular (*cis*) inteins and *trans*‐splicing split inteins have enabled many applications in protein engineering, biotechnology and chemical biology due to their reactive acyl intermediates in the splicing pathway (see below) and their unique ways of protein backbone ligation and cleavage. Well‐splicing split inteins continue to be in urgent demand to fit the diverse requirements for engineering proteins of interest, such as size and sequence composition, *trans*‐splicing rate and yields, solubility and extein sequence tolerance.

**Figure 1:**
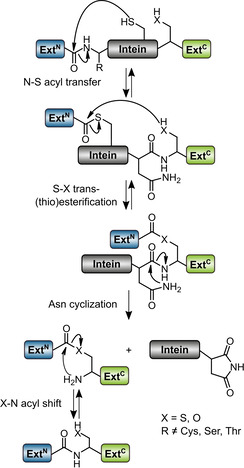
Protein splicing mechanism of class 3 inteins. The difference from class 1 inteins is the use of an internal cysteine for the first branched intermediate instead of a cysteine or serine at the N terminus of the intein. Class 2 inteins show only one branched intermediate, which is formed through direct attack of the +1 cysteine at the first residue of the C‐extein on the N‐terminal scissile bond. Note, the new AceL NrdHF intein described in this work is a split intein, in which the N‐ and C‐terminal fragments of the intein domain first have to associate and fold into the active intein (not shown here).

Inteins can be regarded as single turnover enzymes. They catalyze a set of acyl rearrangement and bond cleavage reactions with contributions from a few highly and well conserved residues that reside in sequence block motifs referred to as N1, N3, C2, and C1.^[3]^ Three types of splicing mechanisms have been identified so far, according to which both cis and split inteins are grouped into classes. Class 1 and 2 inteins rearrange the N‐terminal scissile bond into branched intermediates of the polypeptide chain with branch positions at the first residue of the intein or the first residue of the C‐extein, respectively. The more recently discovered and less well‐studied class 3 inteins, by contrast, form an internally branched intermediate in the first step of the pathway. As shown in Figure 1, the internal motif C2 : 4 cysteine performs a nucleophilic attack at the N‐scissile bond to give the branched thioester intermediate. In the second step, the attack of the side chain of the first residue of the C‐extein, residue Cys+1, Ser+1 or Thr+1, leads to a second branched intermediate by a transesterification reaction. The last intein‐catalyzed step then involves an asparagine cyclization of the conserved ultimate residue of the intein (motif C1 : 7) to cleave the C‐scissile bond and thereby separate the intein from the exteins. Finally, the (thio‐)ester bond between N‐ and C‐exteins spontaneously rearranges in a S−N (or O−N) acyl shift to form a native peptide bond and constitute the mature host protein.^[4]^

Newly identified intein sequences are usually categorized into one of the classes on the basis of conserved sequence features in their motifs. For example, class 2 inteins lack a residue with nucleophilic side chain at the motif N1 : 1 position. Class 3 inteins were found to contain a signature typical WCT triplet, representing a tryptophan at the motif N3 : 12, a cysteine as the branch position at motif C2 : 4 and a threonine at motif C1 : 5.^[4a]^ While the cysteine and threonine are directly involved in catalysis of the splice reaction, the tryptophan is thought to be of structural importance.^[4b]^

In addition to the mechanistic distinctions of the splicing pathways of class 1, 2 and 3 inteins, many more subtle differences can be found between various inteins with respect to how these reactions are catalyzed and how important the contribution of a certain residue is.^[5]^ Further plasticity in the protein splicing pathway is found on the level of the kinetic control and coordination of catalyzed steps.^[6]^

Inteins fold in the HINT (Hedgehog/intein like) structure, which is conserved for all classes of inteins. Crystal structures have illustrated how the key catalytic residues are positioned in close proximity and helped explain how inteins facilitate catalysis and link the N‐ and C‐exteins with minor conformational changes. The internal branch point cysteine of the WCT triplet is structurally located between the N‐ and C‐scissile bonds. However, the only so far reported crystal structure of a class 3 intein shows only the excised intein and therefore no structural information on the extein residues.^[7]^

Inteins can also contain a homing endonuclease (HEN)[^3b^, ^8^] as an inserted domain or consist of two fragments in form of a split intein that have to associate and fold into the active intein before protein *trans*‐splicing (PTS) occurs. Split inteins are naturally found and can also be created artificially in many cases.[^2a^, ^9^] Intriguingly, no split class 2 or class 3 inteins have been reported so far.

We identified from metagenomic sequence data the AceL NrdHF intein as the first split class 3 split intein. The intein exhibits several unusual sequence features. We report a biochemical, biophysical, structural and mutagenesis study. Our results further expand the view of inteins being a very diverse group of proteins with malleable properties and mechanisms. Furthermore, they suggest that the AceL NrdHF holds potential to be an important addition to the intein toolbox for protein ligation and modification purposes.

## Results and Discussion

### Class 3 intein types

**Figure 2:**
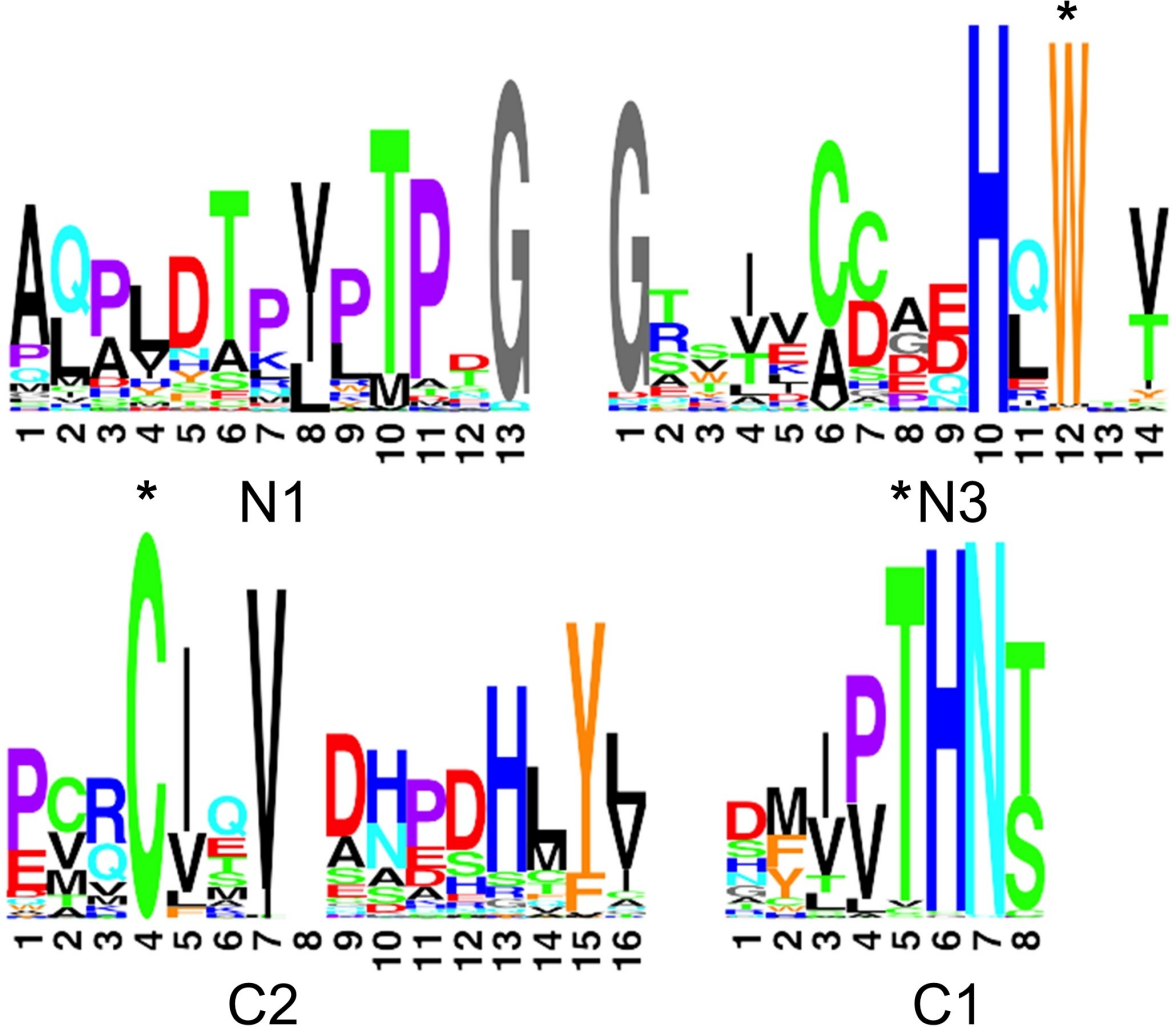
Sequence motifs of class 3 intein's active site. Sequence logo representation of active‐site motifs shown as in ref. [3b]. Briefly, the height of each amino acid and position is proportional to their conservation, after the sequences have been weighted and frequencies adjusted to the expected amino acid composition. Amino acids are colored by their chemical and physical properties. The positions of the class‐3 WCT triplet motif are marked by asterisks. Note that position 8 of the C2 motif is an insert to align it with this motif in class 1 inteins.^[7]^

Analyzing intein sequences in the NCBI database of non‐redundant proteins (nr) we identified a set of 1701 class 3 inteins as inteins with no cysteine in their N‐end (motif C1 : 1) and a cysteine in their motif C2 : 4 position. The vast majority (82.5 %) of these inteins, similarly to class 1 inteins, contained LAGLI‐DADG type homing endonuclease motifs.^[3b]^ Such inteins were suggested to constitutively splice and be mobile selfish genetic elements.^[7]^ All of these inteins are cis‐splicing inteins, suggesting that split class 3 inteins are very rare. Identifying a very large data set of class 3 inteins together with careful sequence analyses enabled us to update their conserved and diverged sequence features in fine detail. Figure 2 shows an updated version^[4a]^ of the conserved sequence motifs from this group. The class 3 WCT signature triplet had few deviations of the eponymous amino acid triplet, with rare deviations to threonine C1 : 5 and tryptophan N3 : 12 (4.6 and 6.1 %, respectively; see Table S1 in the Supporting Information for full details).

### Sequence identification of a novel class 3 split intein

**Figure 3:**
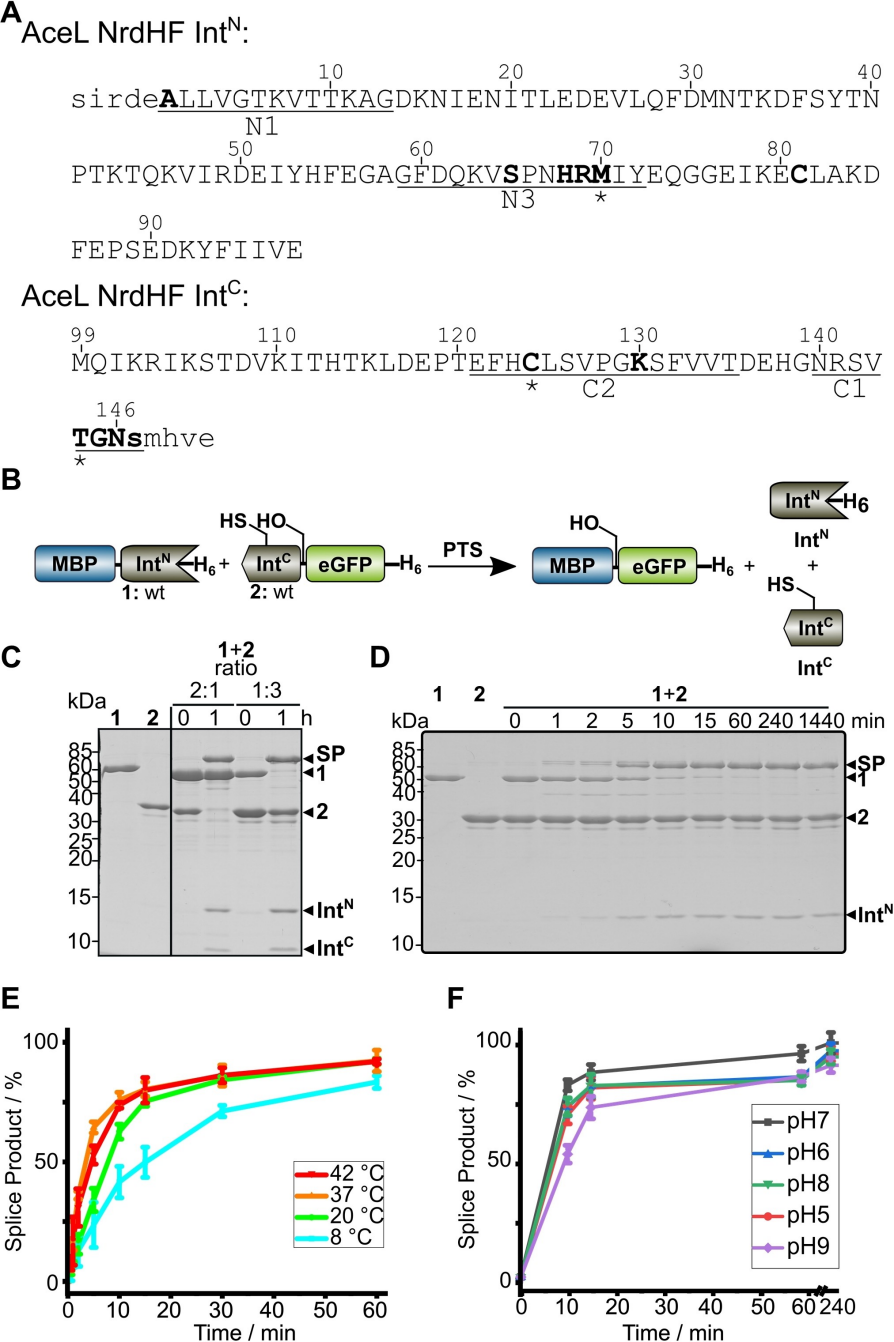
The AceL NrdHF intein and its protein *trans*‐splicing activity. A) Sequences of the Int^N^ and Int^C^ fragments with five extein residues each (in lower case). Block motifs (underlined) and residues mutated in this study (bold) are highlighted. B) Scheme of a PTS assay with model proteins. C) SDS‐PAGE analysis of PTS reactions at 37 °C with one intein precursor in molar excess over the other. A representative Coomassie‐stained gel is shown. D) Analysis as in (C) of the reaction over time with **1** at 5 μM and **2** at 25 μM. E) Time course of PTS for different temperatures determined by densitometric analysis of the data as shown in (D) for 37 °C. F) pH dependence of PTS at 37 °C. Error bars indicate standard deviations. Calculated molecular weights are: **1**: 55.8 kDa; **2**: 34.0 kDa; SP: 72.3 kDa; Int^N^: 12.2 kDa; Int^C^: 5.5 kDa. SP=splice product (MBP‐eGFP).

We analyzed metagenomic data sets from the Antarctic Ace Lake^[10]^ and identified a putative novel class 3 split intein. The intein coding regions are located in a 12.2 kb metagenomic contig consisting of 17 genes that by similarity and organization might originate from a bacteriophage. The sequence was found to be embedded in the *nrdHF* gene encoding for a class Ib nucleoside diphosphate reductase (RNR) beta subunit, splitting it into two genes in a fractured‐gene organization with an intervening GIY‐YIG type putative homing endonuclease.^[11]^ The split intein was named AceL NrdHF and has a total of 146 amino acids (aa), with a 98‐aa Int^N^ and a 48‐aa Int^C^ part (Figure 3A). The split site is located at a typical breakpoint of split inteins where a homing endonuclease (HEN) domain is present in many inteins.[^3b^, ^8^] Based on its sequence features and similarity to other inteins we predicted the AceL NrdHF intein to be the first split class 3 intein. In particular, no nucleophilic residue was found at position 1, which is an alanine, and a cysteine was located in the motif C2 as a prerequisite for the characteristic internal branched intermediate. However, due to poor sequence conservation uncertainty remained whether the cysteine was at the correct position for this role. Structural modeling using the Phyre2 server^[12]^ failed to properly align the motif C2 and C1 residues with existing intein structures. Furthermore, the sequence was at odds with other expectations for a class 3 intein. In particular, the usually strictly conserved tryptophan from the WCT triplet signature was missing. With a methionine found at the motif N3 : 12 position the intein instead exhibits an unusual MCT triplet (residues 70, 124 and 144). Furthermore, the two conserved histidine residues in motifs C2 : 13 and C1 : 6, which are important for catalysis of the asparagine cyclization, are missing in the AceL NrdHF sequence.

### Biochemical and biophysical characterization and functional classification as a split class 3 intein

For functional characterization the intein fragments of the AceL NrdHF intein were obtained as synthetic gene fragments and cloned in fusion with gene fragments encoding tag sequences and model proteins. Five native extein residues were kept as N‐terminally (SIRDE) and C‐terminally (SMHVE) flanking regions of the intein, respectively. Following expression in *Escherichia coli* the precursor proteins MBP‐Int^N^‐H_6_ (**1**) and Int^C^‐eGFP‐H_6_ (**2**) were purified by affinity chromatography (eGFP=enhanced green fluorescent protein, MBP=maltose binding protein). To assay protein *trans*‐splicing activity according to the scheme in Figure 3B, **1** and **2** were mixed, reaction aliquots were removed at different time points, immediately boiled in SDS sample buffer to stop the reaction and analyzed by SDS‐PAGE. Figure 3C shows that the intein was indeed active and produced the expected protein products. Both precursors spliced to virtual completion in 1 h at 37 °C when the complementary fragment was present in excess. LC‐MS analysis of the reaction mixture at 37 °C and in‐gel digestion of the protein band of the splice product MBP‐eGFP further verified protein splicing and the correct prediction of both splice sites (Figure S1). The highest rate of splicing was found at 37 °C and pH 7. Fitting of the data to a pseudo‐first‐order equation revealed a rate constant *k*=(30.9±1.9) 10^−4^ s^−1^ corresponding to a *t*_1/2_ of ∼3.8 min (Figure 3D). Efficient yields within acceptable periods of time were also observed over a broad range of temperatures and pH values (Figure 3E and F). The observed temperature optimum at 37 °C is noteworthy, as the temperature at the sampling site of the environmental probe in the Antarctic Ace Lake was 0.4–2.7 °C. We previously characterized the AceL TerL intein from the same location and observed a clear adaption of the intein's optimal activity to the cold temperature.^[2c]^ It seems no similar evolutionary pressure was on the present AceL NrdHF intein for further adaptation, possibly because its activity at lower temperatures was high enough.

N‐ and C‐terminal cleavage reactions, known as typical side reactions in protein splicing, were at low and negligible levels, or not even detectable. Of note, the occurrence of traces of a protein band at ∼42 kDa corresponding to free MBP as the N‐cleavage product in the first minutes of the reaction was only of transient nature (Figure 3B). Its subsequent disappearance at later time points shows that the cleavage reaction occurred only during sample preparation of the removed aliquot for SDS‐PAGE analysis, most likely through the β‐mercaptoethanol (β‐ME) in the SDS buffer acting as a nucleophile to cleave the thioester intermediate. Consistent with this interpretation, another faint additional band can be detected with a similar transient appearance, migrating just above the splice product (SP) in the SDS‐PAGE gel shown in Figure 3B. Given that thioesters should be cleaved efficiently by β‐ME we believe this band represents the second (oxoester) branched intermediate, which is in chemical equilibrium with the first thioester intermediate. The transient build‐up of the branched intermediate has previously been observed for other inteins and is indicative of asparagine cyclization as the last step of protein splicing being rate determining for the entire splicing pathway.[^6a^, ^6c^, ^13^]

**Figure 4:**
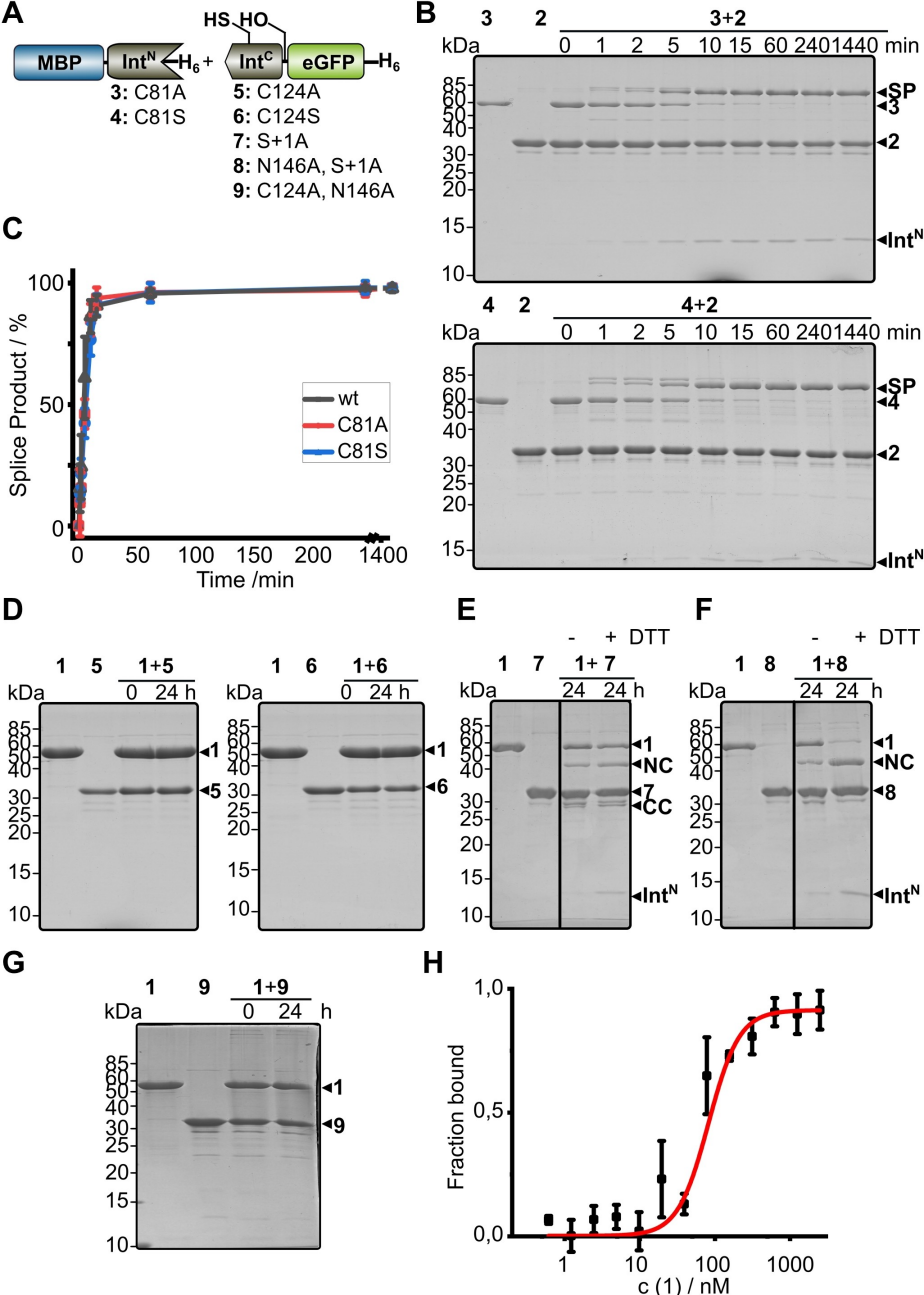
Analysis of the nature and coordination of the splicing mechanism. A) Intein constructs. B), D)–G) End‐point (Int^N^ and Int^C^ precursors=10 μM) or time‐dependent analyses (Int^N^ precursors=5 μM; Int^C^ precursors=25 μM) of the indicated protein *trans*‐splicing reactions at 37 °C using Coomassie‐stained SDS‐PAGE gels. Int^N^ and Int^C^ precursor proteins were mixed to start the reaction. C) Time course of the indicated reactions determined by densitometric analysis of the data shown in (B) and Figure 3D. Calculated molecular weights are similar to those given for the wild type (wt) constructs in Figure 3. SP=splice product; CC=C‐terminal cleavage product; NC=N‐terminal cleavage product. H) Determination of the dissociation constant between inactivated **9** and **1** by using microscale thermophoresis (MST). eGFP served as the fluorophore required for MST measurements. Construct **9** (8.8 nM) was mixed with **1** in a dilution series from 2500 to 0.61 nM. Calculated *K*_d_=(82.2±9.1) nM. Error bars indicate standard deviations.

We then investigated whether the AceL NrdHF intein indeed follows a class 3 splicing mechanism. Besides the above‐mentioned C124 in the putative motif C2 motif of the Int^C^ fragment, a single cysteine (C81) is located in the Int^N^ fragment at a non‐conserved position (Figure 3A). Mutation of the non‐conserved cysteine within the Int^N^ precursor in a C81A (construct **3**) or C81S mutant (**4**) did not affect splice product formation compared to the wildtype (wt) intein (Figure 4A–C), suggesting this residue is unrelated to the splicing mechanism. In contrast, both the C124A and the C124S mutations in the Int^C^ precursor (constructs **5** and **6**, respectively) resulted in a complete loss of activity (Figure 4D), consistent with an essential role of this residue to facilitate the first N–S acyl transfer in splicing and act as the first branch point position. Interestingly, C‐cleavage activity was also blocked by these mutations, suggesting a tight functional coupling of the asparagine cyclization step to previous steps in the pathway. To further investigate the coordination between the individual steps, we generated the S+1A mutant of the Int^C^ precursor protein (construct **7**) to block formation of the second branched intermediate formed at the first C‐extein residue. This mutant was also inactive in splicing, as expected, and showed partial N cleavage of the presumed first branched intermediate involving the C124 thioester and partial C cleavage (Figure 4E). The N‐cleavage reaction could be enhanced only slightly by additionally adding DTT at high concentrations (50 mM) as a nucleophile to intercept the thioester intermediate (Figure 4E). These findings suggest that the equilibrium of the initial N−S acyl transfer is largely on the side of the peptide bond when the transesterification to the second branched intermediate is blocked by mutation. Only when we additionally mutated the catalytic asparagine at the ultimate motif C1 : 7 position of the intein to alanine (N146A; construct **8**), nearly quantitative N cleavage could be enforced with 50 mM DTT (Figure 4F), possibly as a result of the additional structural changes in the active site that might have impacted the coordination of the first two steps. As expected, C‐cleavage activity was completely abolished in the double mutant **8** (S+1A, N146A). Together, these findings confirmed a class 3 splicing mechanism for the AceL NrdHF intein. The equilibrium of the initial N−S acyl transfer on the side of the peptide bond and the tight coupling of asparagine cyclization to the previous steps explain the observed efficient suppression of off‐pathway reactions during protein splicing.^[4c]^

To study the affinity between the fragments of the AceL NrdHF intein, we used inactivated Int^C^ precursor protein with the C124A and N146A mutations (construct **9**), to separate intein fragment association from protein splicing steps. By using microscale thermophoresis (MST) we determined a dissociation constant of 82±9 nM (Figure 4G and H).

### AceL NrdHF‐1‐1 intein crystal structure

**Figure 5:**
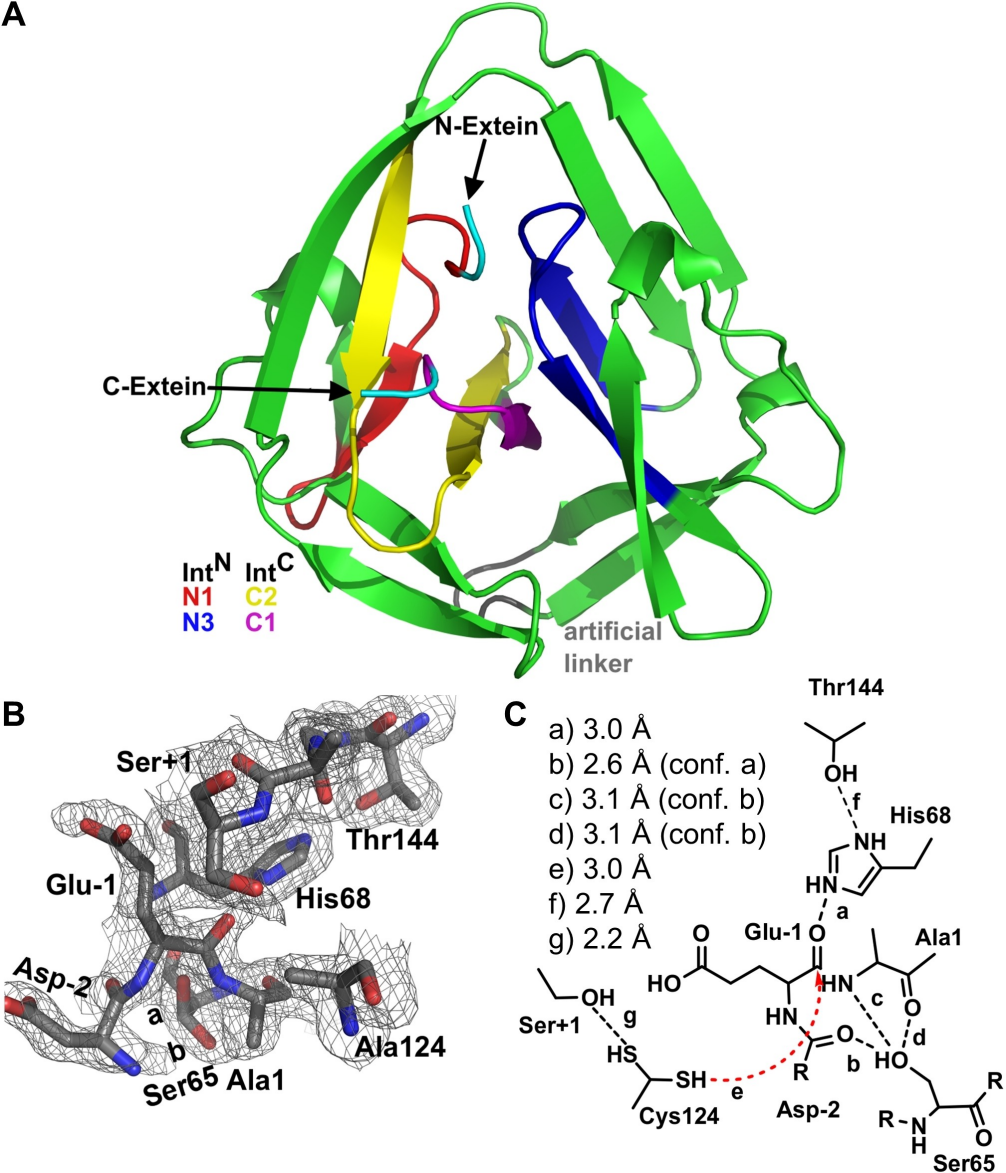
Crystal structure of the AceL NrdHF intein at 1.9 Å. A) Structure of the crystallized C124 A/N146 A precursor. Block motifs are highlighted in different colors. B) Catalytic center of the intein shown in an electron density map contoured at the 1.0 *σ* level. Ser65 (B:7) shows two distinct conformations. C) Illustration of the interactions of the key catalytic residues with distances discussed in the main text. Cys124 was modeled into the structure leading to two possible conformations.

To better understand the mechanism and unusual sequence features of the novel AceL NrdHF intein, we determined its structure. The only reported crystal structure of a class 3 intein so far is of the spliced *Msm* DnaBi1 intein, a typical class 3 *cis*‐intein.^[7]^ To increase the chances of capturing the intein with active site residues in relevant conformations for splicing we decided to crystallize the precursor and fused the AceL NrdHF fragments with inactivating C124A and N146A mutations. Five native flanking extein residues were included at the N‐ and C‐terminal sides, respectively. Expression of the fused intein domain in *E. coli* without the inactivating mutations resulted in quantitative splicing with no detectable precursor protein remaining, thus suggesting that the artificial cis‐arrangement did not have an impact on folding and function (Figure S2). We determined the structure of the AceL NrdHF precursor at a resolution of 1.9 Å (see Figures S3 and S4 and Table S2 for sample preparation and general crystal data). The intein adopted the typical HINT fold with the expected localization of the conserved motifs in and around the active site (Figure 5A). The asymmetric unit contained two protein chains that form an inter‐chain antiparallel β‐sheet involving residues A12–K15 and come in close proximity at residues E140–G142. However, these contacts are far away from the active site and did not seem to influence the structure of the intein. The conformations of both chains are highly similar with a root means square deviation (rmsd) of 0.223 Å over all atoms (Figure S4A).^[14]^ Electron density can be observed only for the two N‐ and two C‐terminally flanking residues in chain A as well as two N‐ and three C‐terminal extein residues in chain B, suggesting that extein residues further outside were disordered. All intein residues showed electron density including the three residues (GSH) that were inserted upon fusion of the Int^N^ and Int^C^ fragments.

### Structure‐based mutation analysis of motif residues at the N‐terminal splicing junction and the WCT triplet

We analyzed the active site of the intein with regard to Cys124, which was assumed as the internal branch point position for the first intermediate based on the above‐mentioned initial mutagenesis study. Cys124, in the structure mutated to alanine, was indeed located in the active site at the expected position within the motif C2 motif to allow interactions with both splice junctions and to act as the branch point residue (Figure 5B). This position is equivalent to a catalytic C2 : 4 aspartate residue found in many class I inteins.^[15]^ We modeled the cysteine side chain back in and found that two possible rotamer conformations fit well to its expected orientation towards either the N‐scissile bond or towards the Ser+1 at the C‐terminal splice junction. The distance of the thiol side chain to the carbonyl carbon of the N‐scissile bond would be only 3.0 Å, sufficiently short to trigger the initial N−S acyl transfer without additional larger conformation changes (Figure 5C).

**Figure 6:**
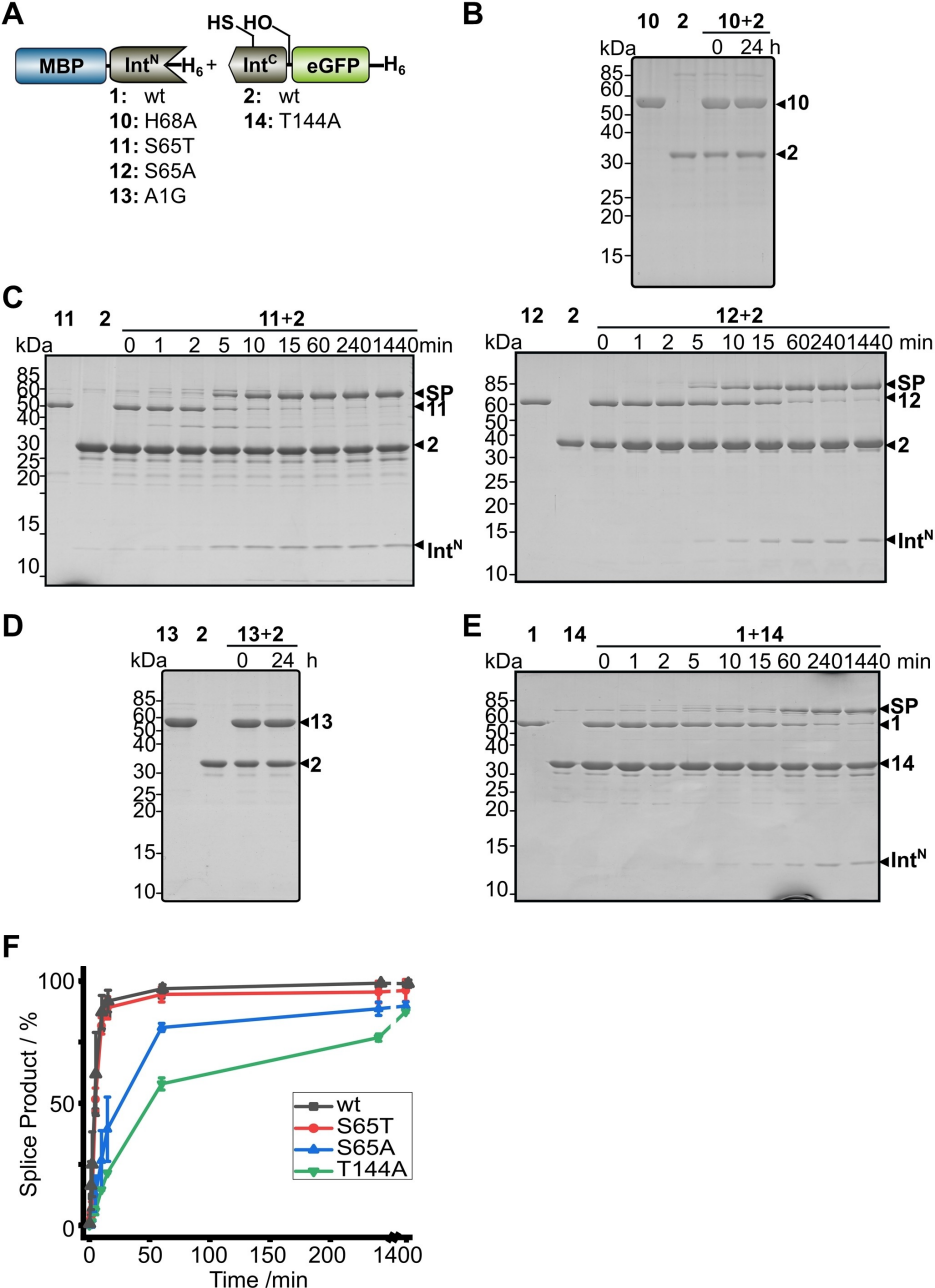
Mutational analysis of the N‐terminal splice junction. A) Intein constructs. B)–E) End‐point (Int^N^ and Int^C^ precursors=10 μM) or time‐dependent analyses (Int^N^ precursors=5 μM; Int^C^ precursors=25 μM) of the indicated protein *trans*‐splicing reactions at 37 °C using Coomassie‐stained SDS‐PAGE gels. Int^N^ and Int^C^ precursor proteins were mixed to start the reaction. F) Time courses of the PTS reactions with the indicated mutants determined by densitometric analysis of the data shown in (C, E) and Figure 3D. Calculated molecular weights are similar to those given for the wt constructs in Figure 3. Error bars indicate standard deviations. SP=splice product.

We then studied the importance of catalytic residues on the splicing activity at the N‐terminal splice junction (Figure 6). The highly conserved motif N3 : 10 histidine (His68) is in spatial hydrogen‐bond distance to the carbonyl group of the Glu1 residue. The key role of the histidine appears to be the distortion around the N‐scissile bond, consistent with other crystal structures of class 1 inteins and biochemical studies.[^5d^, ^16^] Mutation of the residue in a H68A Int^N^ precursor mutant (**10**) completely abolished protein *trans*‐splicing, indicating its critical role (Figure 6B). The adopted unusual conformation at the N‐terminal splice junction confers dihedral angles deviating from a trans peptide bond, which seemingly are further stabilized by the motif N3 : 7 serine residue (S65) that is found populating two side chain conformations. In one rotamer it potentially hydrogen bonds (3.2 Å) with the carbonyl oxygen of the motif N1 : 1 alanine residue and in the other conformation it is in potential hydrogen bond distance to both the N‐scissile bond amide nitrogen (2.9 Å) as well as to the −2 carbonyl oxygen (3.3 Å; Figure 5C). Various roles have been suggested for the N3 : 7 residue in activation of the N‐terminal splice junction in all classes of inteins.[^4a^, ^4b^, ^17^] Class 3 inteins in general do not show a conserved residue at this position (Figure 2), and have been functionally characterized with a N3 : 7 glycine or aspartate.[^4a^, ^7^, ^17a^] A functional role as suggested here for the N3 : 7 serine has previously been described as a molecular spring load mechanism, that is typical for class 1 inteins usually showing an N3 : 7 threonine in a TxxH motif.^[17b]^ Ser65 would assist the N3 : 10 histidine in positioning and distorting the N‐scissile bond and might be involved in stabilizing the oxyanion intermediate of the N−S acyl transfer reaction. This analogy to class 1 inteins is further underlined by analysis of a S65T mutant (**11**), which exhibited indiscernible protein *trans*‐splicing activities when compared to the wt intein (Figure 6C). A S65A mutant (**12**) was still active in protein *trans*‐splicing but its 4‐fold impaired rate demonstrates the importance of Ser65 (Figure 6C). Interestingly, the twisted arrangement at the N‐scissile bond, with the distortion leading to the coordination of the −1 carbonyl oxygen with N3 : 10 histidine, has only been observed so far in two intein structures[^5d^, ^18^] and the present case is the first example for a class 3 intein. This is noteworthy because as a class 3 intein the AceL NrdHF intein employs the motif C2 thiol instead of the immediately adjacent side chain of the motif N1 : 1 residue at position 1 of the intein for the nucleophilic attack. The distortion primes the N‐scissile bond for the attack from the backside, with regard to the position of the motif N3 : 12 histidine, as previously predicted by the observed gradual twisting of the N‐scissile bond in a series of mutant structures of the *Ssp* DnaB intein.^[5d]^ Together, these findings suggest the structure has captured the AceL NrdHF intein in the conformation competent for the first step of N−S acyl transfer.

To further test the idea of a conformationally distorted N‐scissile bond, we introduced the mutation A1G into the Int^N^ precursor (construct **13**), because glycine with its higher flexibility can adopt a wider range of dihedral angles. Indeed, the protein *trans*‐splicing activity was completely abolished (Figure 6D), further corroborating the requirement for a strained arrangement at the N‐terminal splice junction.

The motif C1 : 5 threonine (Thr144) of the WCT triplet is positioned in hydrogen bonding distance of 2.6 Å to the motif N3 : 10 histidine (His68; Figure 5C) indicating it affixes His68 in its critical conformation and possibly helps to activate it through electronic polarization. This contribution is important but not crucial, because we found that a T144A mutant (**14**) still showed protein *trans*‐splicing, albeit with an about tenfold reduced rate (Figure 6E). As the high conservation of this position with threonine is only observed for class 3 inteins, it represents a typical class 3 characteristic of the AceL NrdHF intein.

**Figure 7:**
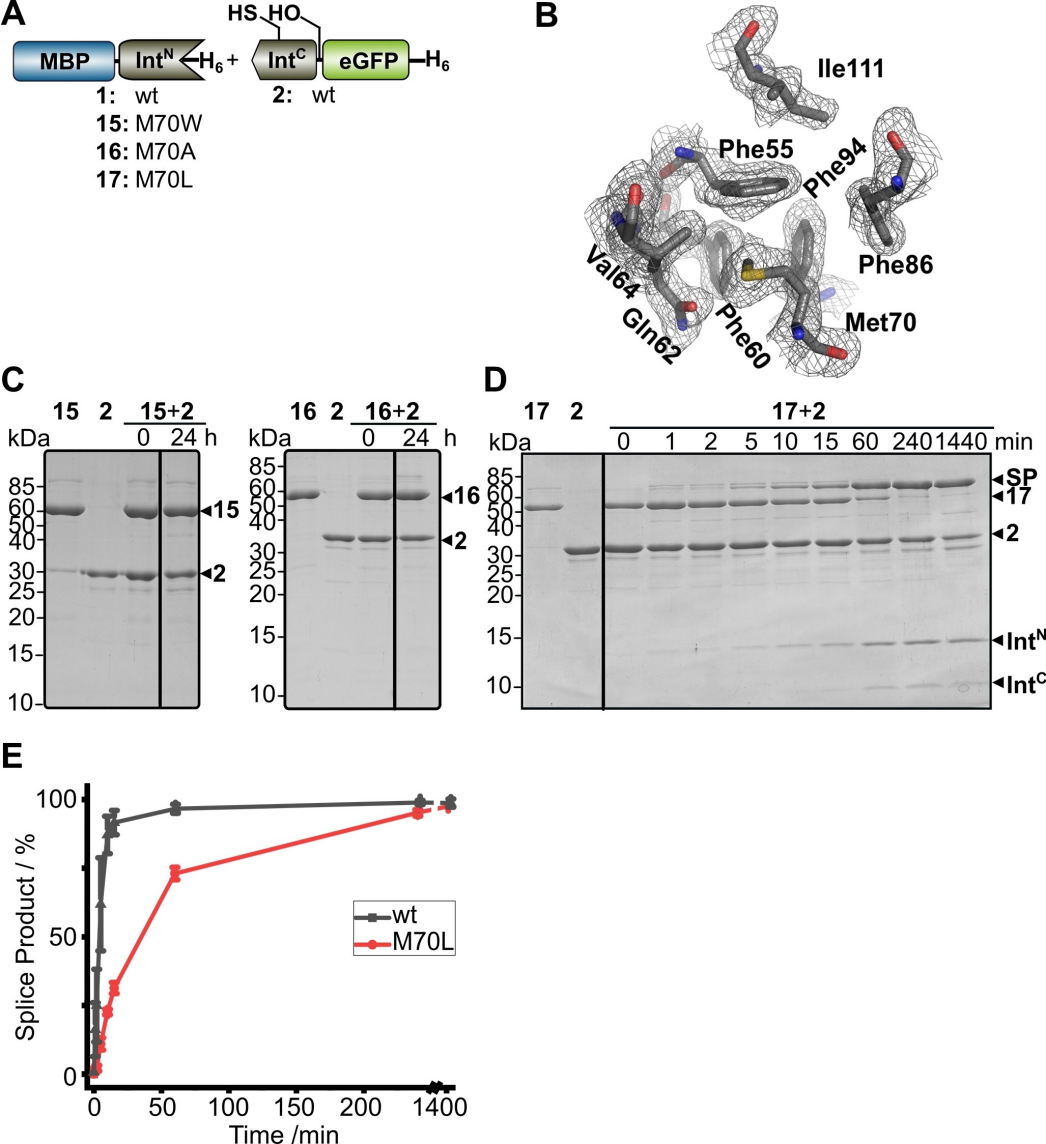
Mutational analysis of the N3 : 12 position. A) Intein constructs. B) Molecular contacts of Met70 in the hydrophobic core of the intein shown in an electron density map contoured at the 1.0 *σ* level. C), D) End‐point (Int^N^ and Int^C^ precursors=10 μM) or time‐dependent analyses (Int^N^ precursors=5 μM; Int^C^ precursors=25 μM) of the indicated protein *trans*‐splicing reactions at 37 °C using Coomassie‐stained SDS‐PAGE gels. Int^N^ and Int^C^ precursor proteins were mixed to start the reaction. E) Time course of the reaction of **17** and **2** determined by densitometric analysis of the data shown in (C, E) and compared to the data of the wt intein from Figure 3D). Calculated molecular weights are similar to those given for the wt constructs in Figure 3. Error bars indicate standard deviations. SP=splice product.

Finally, we looked into the most obvious deviation of the AceL NrdHF intein from a typical class 3 intein, namely substitution of the highly conserved tryptophan in the WCT triplet to methionine at the motif N3 : 12 position (Figure 7). Tori et al. showed the structural importance of this residue for the class 3 MP‐Be DnaB intein. Substitutions to smaller hydrophobic residues were tolerated poorly to fairly well.^[4a]^ In contrast, class 1 inteins typically show a large, hydrophobic residue at this position (Phe, Leu, Met, Trp, Val or Ile).^[4a]^ Our crystal structure shows the M70 side chain centrally located in the hydrophobic core as expected (Figure 7B). A M70W mutation (construct **15**) completely impaired all intein activity (Figure 7C, left), as did substitution with the other aromatic residues (data not shown). Interestingly, a M70A mutant (**16**) was also completely inactive (Figure 7C, right), underlining the importance of this position for a fine‐tuned folding of the intein. In contrast, a M70L mutation (**17**) was tolerated and resulted in a sevenfold rate reduction in protein *trans*‐splicing (Figure 7D and E). Altogether, with regard to the first position of the WCT triplet (motif N3 : 12), the AceL NrdHF intein behaved more like a class 1 than like previously examined class 3 inteins.

### Structure‐based mutation analysis of motif residues at the C‐terminal splicing junction

**Figure 8:**
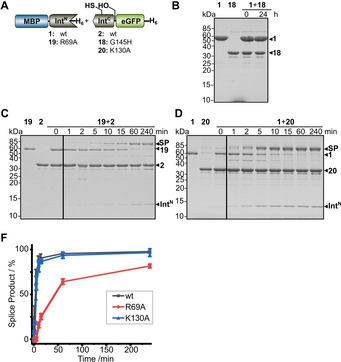
Mutational analysis of the C‐terminal splice junction. A) Intein constructs. B)–D) End‐point (Int^N^ and Int^C^ precursors=10 μM) or time‐dependent analyses (Int^N^ precursors=5 μM; Int^C^ precursors=25 μM) of the indicated protein *trans*‐splicing reactions at 37 °C using Coomassie‐stained SDS‐PAGE gels. Int^N^ and Int^C^ precursor proteins were mixed to start the reaction. F) Time courses of PTS reactions with the indicated mutants determined by densitometric analysis of the data shown in (C, D) and compared to the data for the wt intein taken from Figure 3D. Calculated molecular weights are similar to those given for the wt constructs in Figure 3. Error bars indicate standard deviations. SP=splice product.

Another unusual sequence feature of the AceL NrdHF intein is the simultaneous absence of both catalytic histidine residues in the C2 and C1 motifs that usually contribute to the polarization of the asparagine side chain^[19]^ and main chain carbonyl,[^19^, ^20^] respectively, and thereby help in catalysis of asparagine cyclization and cleavage of the C‐scissile bond. Introducing the motif C1 : 6 histidine by a G145H mutation (**18**) impaired all activity in the protein *trans*‐splicing reaction (Figure 8A and B). Structure‐based mutagenesis simulations suggested a steric clash with the side chain of the N3 : 11 arginine Arg69, which is also found in hydrogen bonding distance (2.5 Å) to the C‐scissile carbonyl oxygen and therefore might act in a compensatory mechanism in asparagine cyclization, as was shown for other inteins lacking the block C1 histidine.^[5a]^ In agreement with these considerations, an R69A mutation (construct **19**) significantly slowed down the protein *trans*‐splicing reaction, which, however, still proceeded to virtual completion, suggesting Arg69 is important, but not essential, for catalysis (Figure 8C). In case of the missing motif C2 : 13 histidine the structure revealed that the second half of motif C2 adopts an unusual fold. P128 and G129 (motif C2 residues 9 and 10) induce a kink in the peptide backbone resulting in a different arrangement of the motif C2 residues compared to other intein structures, like motif C2 residues 9–13 of the *Msm* DnaBi1 intein.^[7]^ Thus, the structure explains why the C2 : 13 histidine, which is located C‐terminally relative to the kink, is not conserved. As the best candidate occupying a comparable space, we identified the adjacent Lys130 of the AceL NrdHF intein, which in some rotamer conformations conceivably could activate the asparagine side chain for the cyclization reaction. However, a K130A mutation (**20**) had no measurable effect on protein *trans*‐splicing (Figure 8D). Together, the structure does not fully explain which alternative mechanism, if any, for the polarization of the Asn side chain is in place for the AceL NrdHF intein, possibly also because this side chain is mutated to alanine in the crystallized protein (N146A).

### Extein dependencies

**Figure 9:**
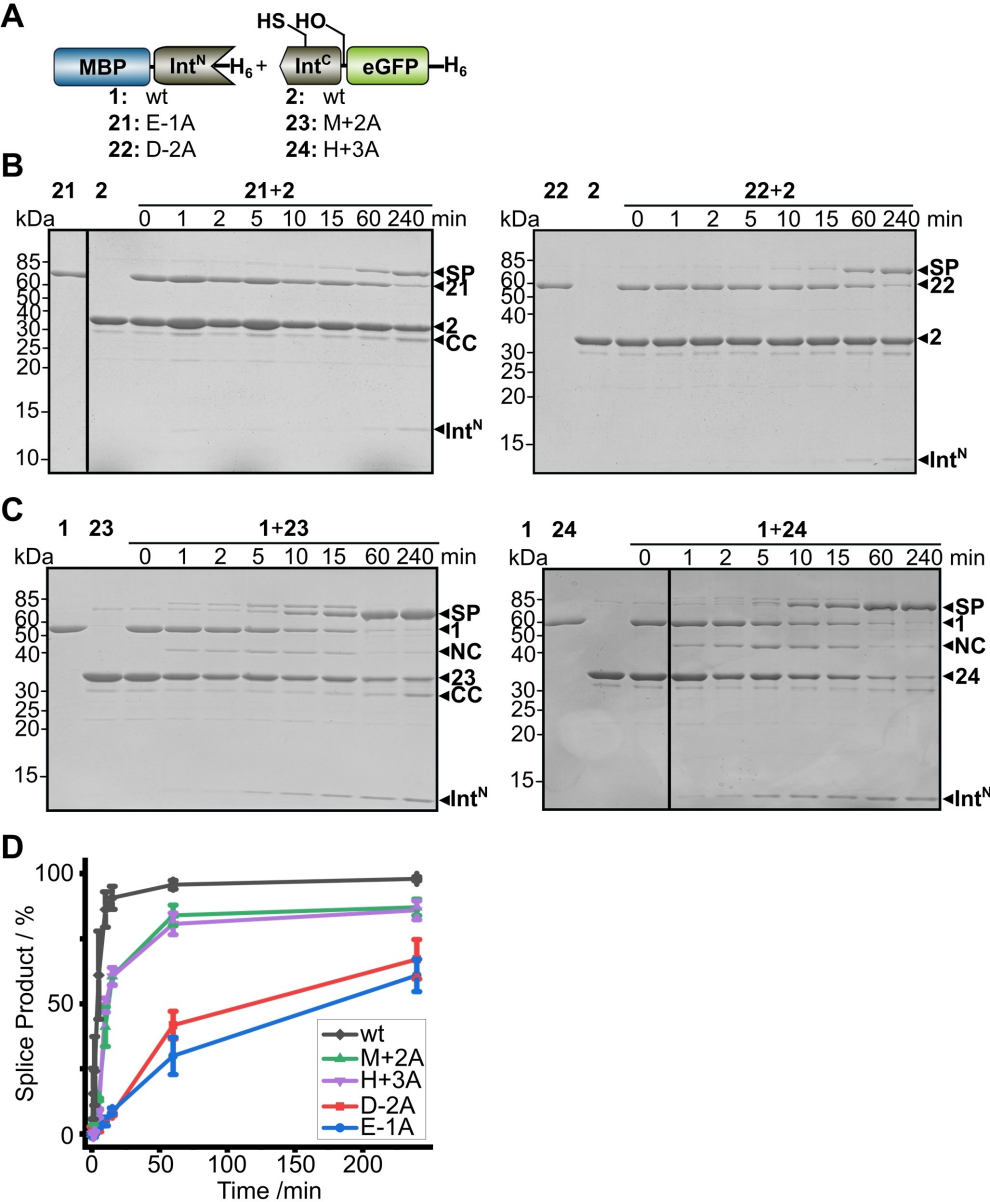
Probing intein tolerance towards extein mutations. A) Intein constructs. B), C) Time‐dependent analyses (Int^N^ precursors=5 μM; Int^C^ precursors=25 μM) of the indicated protein *trans*‐splicing reactions at 37 °C using Coomassie‐stained SDS‐PAGE gels. Int^N^ and Int^C^ precursor proteins were mixed to start the reaction. D) Time courses of PTS reactions with the indicated mutants determined by densitometric analysis of the data shown in (B, C) and compared to the data for the wt intein taken from Figure 3D. Calculated molecular weights are similar to those given for the wt constructs in Figure 3. Error bars indicate standard deviations. SP=splice product; CC=C‐terminal cleavage product; NC=N‐terminal cleavage product.

Inteins do not require a certain sequence at the ligation site except for the single cysteine, serine or threonine residue originating from the first C‐extein residue. They display varying tolerance for the further N‐ and C‐terminally flanking residues and the closest amino acids should have the greatest effect on the intein activity.^[21]^ Mutations E‐1A (construct **21**) and D‐2A (construct **22**) in the Int^N^ precursor of the AceL NrdHF intein still supported splicing in good yields but had substantial effects on the rates of the reactions (Figure 9A, B and D). Mutations M+2A (construct **23**) and H+3A (construct **24**) in the Int^C^ precursor were better tolerated (Figure 9A, C and D). Of note, the mutations in the C‐extein residues also led to the transient appearance of significant amounts of the MBP N‐cleavage product at ∼42 kDa, which suggested, as reasoned above, a more significant build‐up of the branched intermediate(s), in turn indicating a relative decrease of the rate of the asparagine cyclization step. Together, the AceL NrdHF intein displayed a robust tolerance for variations in the extein sequence, in particular on the C‐terminal side.

## Conclusions

We have identified the first naturally split class 3 intein and characterized its properties in protein *trans*‐splicing. Split class 3 inteins seem to be very rare. Furthermore, we analyzed the roles and structural arrangement of several residues important for catalysis, folding and extein dependency. In general, our findings are an important puzzle piece to complement two aspects of the emerging picture of the intein family showing maximal diversity around their common basic concept of function and mechanism. First of all, cis‐inteins with or without HEN domain^[3b]^ and various forms of split inteins,^[2a]^ typically and atypically split,[^2c^, ^22^] can be found in nature. We expect that yet uncovered types of inteins also exist, for example split class 2 inteins or further split variants with atypically short intein fragments that have so far only been artificially created in the laboratory, even though they might be very rare. Second, the contributions of the individual residues properly arranged in the common HINT fold around the N‐ and C‐terminal scissile bonds are associated with a great plasticity between different inteins. The different types of covalently rearranged intermediates, which represent the three intein classes, appears to be the only clear distinction to group them. As proposed previously, it appears reasonable to assume that the unique single‐turnover nature of inteins has helped generate many mechanistic solutions to the same reaction.[^5b^, ^5d^] As optimization of catalytic efficiency appears less important for the cell in case of a single‐turnover enzyme compared to a catalyst required to have high turnover numbers various mechanistic solutions might have led to an evolutionary sufficiently robust performance of the protein splicing reaction.

Inteins in general and split inteins in particular are of great interest in a large variety of applications that rely on their unique properties for the ligation, cleavage or chemical modification of proteins and polypeptide chains. Unfortunately, important parameters of intein and intein fragment precursors, like rate of splicing, solubility and extein dependency, cannot currently be predicted from their primary sequence, thus necessitating individual and elaborate characterization of novel intein candidates.^[23]^ The described novel AceL NrdHF split intein exhibits many very favorable properties that should make it a useful tool for applications, including relatively rapid and nearly quantitative splicing with negligible cleavage activity over a broad range of temperatures and pH conditions, as well as sequence tolerance with regard to the flanking residues, as described in detail in the results section. Of note is our observation that the single cysteine residue in the Int^N^ fragment could be removed by mutagenesis without any measurable loss of activity. This mutated intein variant thus offers advantages over inteins with cysteines in both fragments, because its Int^N^ can be fused to proteins without potential problems associated with thiol side chains, for example those caused by redox interference with other cysteines or even disulfides in the protein of interest. It also qualifies for protein chemical modification via a short cysteine‐containing extein‐tag at the Int^N^, which is first selectively modified using standard thiol bioconjugation and then spliced to the protein of interest. We have recently introduced this approach as the CysTag technology[^13^, ^24^] and even exploited a split intein completely free of cysteines,^[24c]^ however, additional well‐behaved inteins are still sought after to expand its scope and feasibility.

## Experimental Section

**Materials and methods**: Synthetic DNA was ordered from MrGene. Synthetic oligonucleotides were ordered from Biolegio. All plasmids were confirmed by DNA sequencing. Restriction enzymes were purchased from Fermentas. Buffer reagents, antibiotics and media components were purchased from Applichem, Carl Roth, Sigma‐Aldrich and Thermo Scientific. Ni‐NTA agarose was ordered from Cube Biotech. Anti‐Trx antibody was derived from rabbit and purchased from Sigma‐Aldrich. Error bars represent standard deviations from at least three independent repeats.

**Sequence analyses**: All NCBI nr protein sequences related to the smart00306 (HINT‐N) and smart00305 (HINT‐C) conserved domains were retrieved from the CDD server (https://www.ncbi.nlm.nih.gov/cdd/) on 25th June 2020. Redundant sequences with 99–100 % identities were removed from the set using the CD‐hit program.^[15b]^ Using motifs describing class 3 inteins 1701 sequences with complete intein domains that lacked Cys in motif N1 : 1 position and included Cys at motif C2 : 4 position were identified (Table S4). Sequence logos and sequence‐weighted corrected occurrences were calculated as previously described.^[4a]^

Raw sequence reads were retrieved from the NCBI Sequence Read Archive (SRA) depository for project SRP001803 and assembled using pcap.^[25]^ Intein sequences were identified by their block motifs as previously described.^[3b]^ Sequence weights used in sequence logo and WCT triplet occurrence were calculated from conserved sequence motifs of the 1702 class 3 inteins from the NCBI nr and SRA databases analyzed in this work, using position based sequence weights.^[15a]^

**Protein expression and purification**: *E. coli* BL21 Gold (DE3) cells were transformed with the respective expression plasmids (Table S3). Recombinant protein production and purification was basically performed as previously described.^[24c]^ The protein for crystallization was expressed containing a C‐terminal *Ssp* GyrB(1–150)‐CBD tag for protein purification using chitin affinity chromatography followed by DTT (100 mM) induced tag removal (Figure S3),[^5d^, ^26^] and was subjected to additional purifications steps by size exclusion chromatography in 50 mM Tris **⋅** HCl, pH 7.6 (using a HiLoad 75 26/60 Superdex® 75 column; GE Healthcare Life Sciences) and anion exchange chromatography using a NaCl gradient in 20 mM Tris **⋅** HCl, pH 8.0 (on a MonoQ® 4.6/100 PE column; GE Healthcare Life Sciences). Purified protein was concentrated and stored at 4 °C.

**Protein** ***trans*** **‐splicing assay**: PTS was performed at 37 °C if not differently stated. Reactions were started by mixing the N‐ and C‐terminal intein precursor proteins at indicated concentrations in presence of 2 mM TCEP. At indicated time points aliquots were removed and the reaction was stopped by adding 4×SDS‐PAGE loading buffer (500 mM Tris/HCl, 8 % (*w*/*v*) SDS, 40 % (*v*/*v*) glycerine, 20 % (*v*/*v*) β‐mercaptoethanol, 5 mg/L bromophenol blue, pH 6.8) and boiling (95 °C, 5 min).

**Densitometric analysis and determination of rate constants**: Coomassie‐stained bands were analyzed using ImageJ and normalized to molecular weight of the protein the band represents. Normalized intensities were used to calculate the ratio *x* of splice product and precursor protein to determine splice yield as follows.

$$P\left(\%\right)=\frac{\left(100\cdot x\right)}{100+x}\;\left(1\right)$$

Splice product formation was plotted against the time and fitted to the following pseudo‐first‐order equation using Origin2019b.

$$P=P_{0}\left(1-e^{-kt}\right)\;\left(2\right)$$

with *P*=product, time=*t*, *P*_0_=maximum yield and *k*=pseudo‐first‐order rate constant.

**Microscale thermophoresis**: For measurements with a Monolith NT.115 (NanoTemper) splice inactive mutant **9** of the C‐terminal intein precursor was used. Complex partner **1** was diluted in a dilution series from 2500 to 0.61 nM and **9** was added to each sample at a concentration of 8.8 nM. Furthermore, BSA (0.05 %, *w*/*v*) and Tween‐20 (0.1 %, *v*/*v*) were added to avoid aggregation and adsorption to MST capillaries. MST capillaries were loaded with the samples and measurements were performed using 20 % MST power of the IR laser (*λ*=1480 nm) for thermophoresis and 20 % intensity of the blue LED for excitation of the eGFP. Change in fluorescence (*F*_hot_ to *F*_cold_) was plotted against concentrations of **1** and the generated plot was fitted to a four parametric sigmoidal curve using Origin2019b to determine the *K*_d_ value.

**Protein crystallization**: Initial screening was performed using the sitting drop vapor diffusion method in 96 well plate format with commercial sparse matrix screens (JCSG+, Morpheus, PACT Premier, Proplex and Structure, Molecular Dimensions). The crystallization drops consisted of 0.2 μL of protein solution and an equal volume of reservoir solution. All plates were set up using a semi‐automated dispensing robot (Gryphon, Art Robbins Instruments). After initial crystallization conditions were identified, grid screen optimization was performed by hanging‐drop vapor diffusion. Well diffracting crystals grew in 2 μL drops consisting of an equal amount of protein (14 mg/mL in 50 mM Tris**⋅**HCl, pH 7.6) and reservoir solution (0.1 M malonate, imidazole, boric acid, 25 % PEG 3350, 20 % glycerol). Crystals were soaked in reservoir solution containing increasing amounts of NaI (I. 50 mM NaI, II. 200 mM NaI, III. 1 M NaI). All crystals were flash cooled in liquid nitrogen.

**Data collection, processing, phasing and refinement**: Diffraction data was measured at HZB BESSY MX‐14.2 (Berlin, Germany) using a Pilatus 3 S 2M detector at 100 K. The data were integrated and processed using XDSAPP^[27]^ and Mosflm.^[28]^ Initial phases were obtained using Phaser and MR SAD from the Phenix Suite.^[29]^ The model was refined in iterative cycles of manual building in WinCOOT^[30]^ and refinement using phenix refine^[31]^ and refmac5.^[32]^ The entire protein (two molecules in the asymmetric unit (P1) except for four residues at the start and 8 at the end were traced in electron density. Alternate conformations were modeled for V26, T39, I52, S65, C81, M102, T110, I114, and D139 in chain A. In chain B only alternative conformations of D24 and S65 were modeled. The structure was validated using MolProbity^[32]^ and deposited in the Protein Data Bank with accession code 6ZGQ. Full data collection and refinement statistics can be found in Table S2.

**Mass spectrometry**: MS analysis of intact proteins and an in‐gel tryptic digest of the splice product band was carried out as previously described.^[33]^ For mass spectrometry of intact proteins from splice assays the used elution gradient (5–60 % acetonitrile) was applied over 45 min.

## Conflict of interest

The authors declare no conflict of interest.

## Supporting information

As a service to our authors and readers, this journal provides supporting information supplied by the authors. Such materials are peer reviewed and may be re‐organized for online delivery, but are not copy‐edited or typeset. Technical support issues arising from supporting information (other than missing files) should be addressed to the authors.

Supplementary
