# Supplementary material for: Biochemical and Structural Characterization of an Unusual and Naturally Split Class 3 Intein
Source: Chembiochem. 2020 Sep 30;22(2):364–73. doi: 10.1002/cbic.202000509 (PMC7891396; doi:10.1002/cbic.202000509)
Supplement: Supplementary file 1 — Supplementary [file CBIC-22-364-s001.pdf]

ChemBioChem

Supporting Information

Biochemical and Structural Characterization of an Unusual and Naturally Split Class 3 Intein

Simon Hoffmann, Tobias M. E. Terhorst, Rohit K. Singh, Daniel Kümmel, Shmuel Pietrokovski,* and Henning D. Mootz*

Table of contents

Supporting Figures

Figure S1. LC-MS analysis of PTS reaction after 1 h incubation of **1** & **2** at 37 °C. S2

Figure S2. Splicing of an artificially fused cis-AceL NrdHF intein. S3

Figure S3. Protein production for crystallization of the AceL NrdHF intein. S3

Figure S4. Crystal packing and content of the asymmetric unit. S4

Supporting Tables

Table S1: Class 3 inteins WCT triplet occurrences. S5

Table S2: Data collection, processing and refinement statistics of AceL NrdHF intein. S6

Table S3. Expression plasmids and amino acid sequences. S7-8

Table S4. Sequence accession codes for class 3 inteins from NCBI nr database. S9-12

Supporting References S13

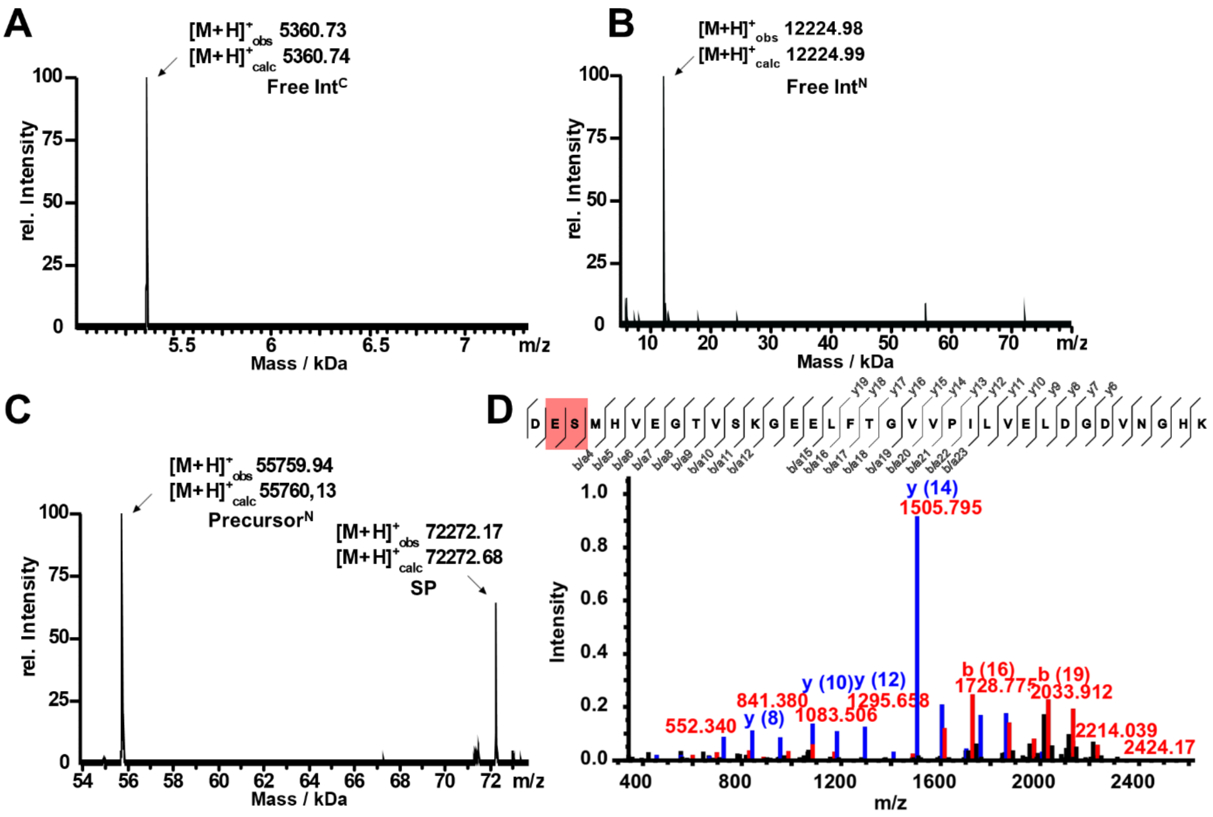

Figure S1. LC-MS analysis of PTS reaction after 1 h incubation of **1** & **2** at 37 °C. Depicted are the calculated masses $[M+H]^+_{calc}$ and the observed masses $[M+H]^+_{obs}$ of (A) free Int^C fragment (the observed mass for the Int^C fragment corresponds to non-hydrolysed Int^C after asparagine cyclization (B) free Int^N fragment (C) N-terminal precursor and splice product (**SP**). (D) MS/MS-mapping of the splice junction in the spliced product (highlighted in red). The corresponding band was excised from a coomassie-stained SDS-PAGE gel and digested using an in-gel digestion protocol before MS analysis. The identified peptide sequence and fragmentation pattern are shown.

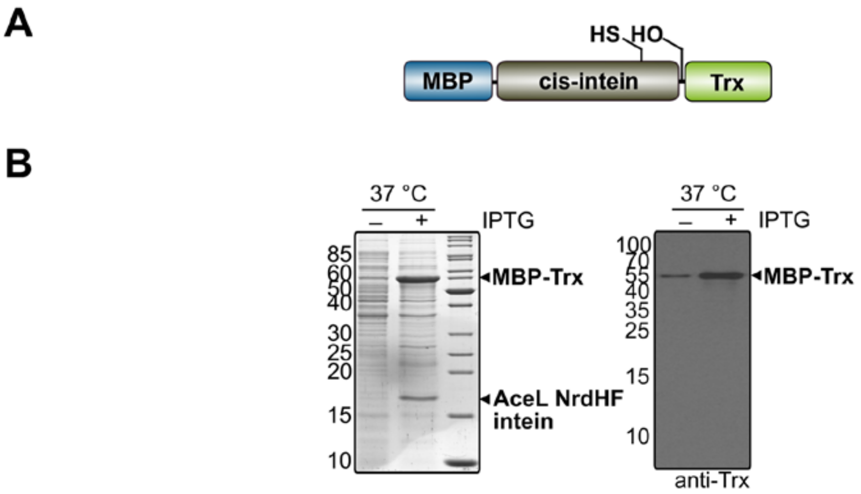

Figure S2. Splicing of an artificially fused cis-AceL NrdHF intein. (A) DNA fragments encoding the fused Int^N and Int^C sequences, connected with a "GSH" linker sequence and 5 native extein residues on each side (SIRDE and SMHVE, respectively) were inserted between gene fragments encoding model proteins maltose binding protein (MBP) and thioredoxin (Trx) to encode an artificial cis intein construct (MBP-MSIRDE-Int^N-GSH-Int^C-SMHVE-Trx). (B) Gene expression in *E. coli* was induced with IPTG and carried out for 3 h at 37 °C. Shown is the analysis of the whole-cell extract using a Coomassie-stained SDS-PAGE gel and Western blot analysis. No unreacted precursor was detected indicating fast splicing kinetics and complete consumption of precursors during splicing in cis. The weak band in the uninduced sample in the western blot analysis suggested a slight leakiness of the promotor.

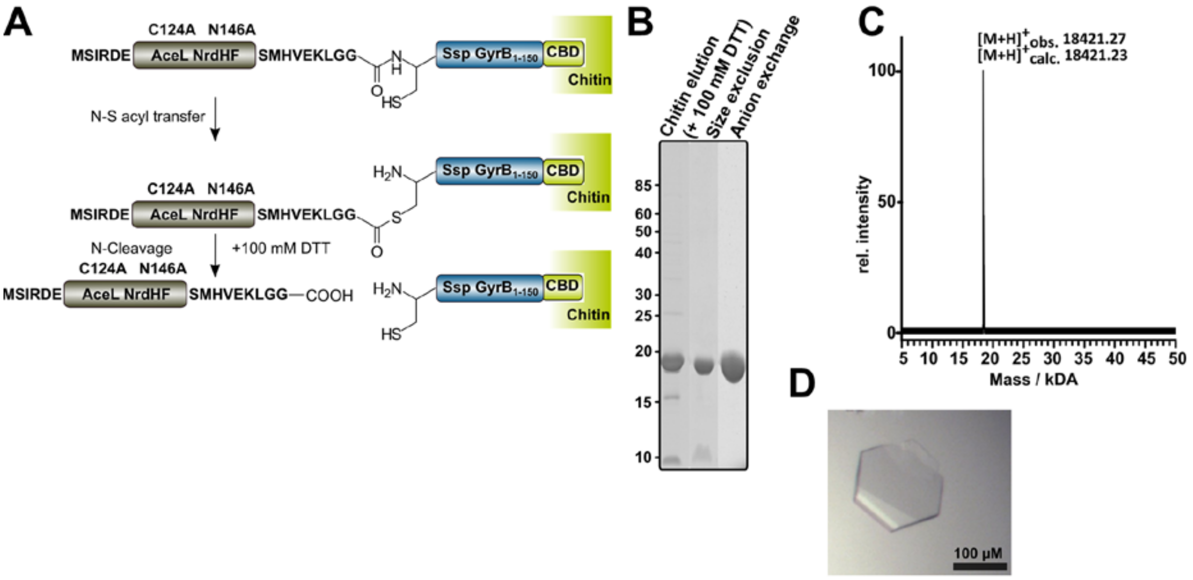

Figure S3. Protein production for crystallization of the AceL NrdHF intein. (A) Schematic representation of protein construct and the purification strategy via a cleavable intein tag as described before.[1] (B) Analysis of protein purification after indicated chromatography steps using a Coomassie-stained SDS-PAGE gel. (C) MS analysis of the protein for crystallization. (D) AceL NrdHF crystal grown in 0.1 M malonate, imidazole, boric acid, 25 % PEG 3350, 20 % Glycerol before NaI soaking.

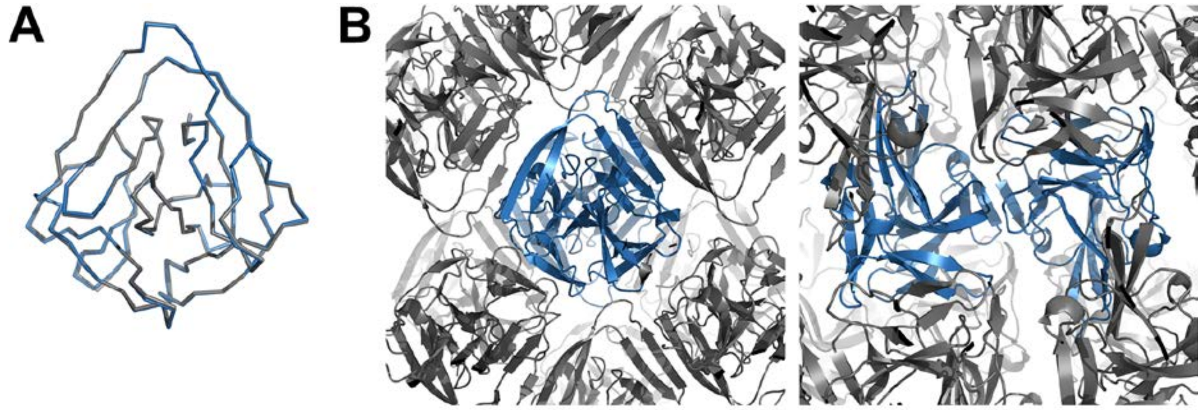

Figure S4. Crystal packing and content of the asymmetric unit. (A) Alignment of the two chains in the asymmetric unit (rmsd: 0.265 Å determined over all atoms using Superpose[2]). (B) Orthogonal representation of the crystal packing. The assembly in the asymetric unit is highlighted in lightblue. Neighboring structures are depicted in grey. The termini of AceL NrdHF are not involved in crystal contacts. The structure was deposited in the Protein Data Bank (PDB: 6ZGQ).

Table S1: Class 3 inteins WCT triplet occurrences. Occurrences of the aa patterns of the WCT triplet (motif positions N3:12, C2:4, C1:5) in the 1702 class 3 inteins analysed in this work. Raw occurrence is the actual observed amount and weighted occurrence is the raw occurrence corrected by the position based sequence weight of each sequence, in percent.

Triplet Sequence	Triplet occurrence	Weighted triplet occurrence / %
WCT	1685	92.06
FCV	7	2.49
MCT	1	1.88
WCS	2	0.82
NCT	3	0.80
WCC	1	0.71
HCT	1	0.59
FCS	1	0.32
WCS	1	0.31

Table S2: Data collection, processing and refinement statistics of AceL NrdHF intein. Values in parentheses refer to highest resolution shell

	Iodide soaked
**Data collection**	
Beamline	HZB BESSY MX-14.2
Detector	Pilatus3 S 2M
Wavelength (Å)	1.5498
Space group	P1
Unique reflections	20988 (1275)
**Cell dimensions**	
a, *b*, *c* (Å)	41.51 45.06 46.28
α, β, γ (°)	65.451 77.508 69.351
Resolution (Å)	41.96 – 1.9 (1.968 – 1.9)
R_{meas}	10.7 (49.0)
I/σ*I*	7.1 (2.0)
CC ½	99.3 (85.6)
Completeness (%)	93.6 (89.3)
Anomalous Completeness	83.7 (79.2)
Iodide sites	7
**Refinement**	
Resolution (Å)	41.96 – 1.9 (1.95 – 1.9)
No. reflections	19917 (1400)
R_{work}/R_{free}	0.230 (0.2880)/0.255 (0.3790)
No. atoms	2721
Protein	2519
Ligand/ion	7
Water	195
Protein residues	307
B-factors (Å^2)	17.7
Protein	14.8
Ligand/ion	51.5
Water	46.2
**R.m.s. deviations**	
Bond lengths (Å)	0.009
Bond angles (°)	1.37
**Ramachandran Plot**	
Favoured Regions	99.67
Allowed Regions	0.33
Outliers	0
MolProbity score	1.67
Molecules/ASU	2
Rotamer outliers	2.8
Clashscore	5

Table S3. Expression plasmids and amino acid sequences.

Protein (**number**)	Expression plasmid	Vector backbone	Sequence
MBP-AceL-NrdHF-1-1-Trx (**Cis**)	pTT63	pMal-c2x	MKTEEGKLVIWINGDKGYNGLAEVGKKFEKDTGIKVTVEHPDKLEEKFPQVAATGDGPDIIFWAHDRFGGYAQSGLLAEITPDKAFQDKLYPFTDAVRYNGKLIAYPIAVEALSLIYNKDLLPNPPKTWEEIPALDKELKAKGKSALMFNLQEPYFTWPLIAADGGYAFKYENGKYDIKDVGVDNAGAKAGLTFLVDLIKNKHMNADTDYSIAEAAFNKGETAMTINGPWAWSNIDTSKVNYGVTVLPTFKGQPSKPFVGVLSAGINAASPNKELAKEFLENYLLTDEGLEAVNKDKPLGAVALKSYEEELAKDPRIAATMENAQKGEIMPNIPQMSAFWYAVRTAVINAASGRQTVDEALKDAQTNSSSNNNNNNNNNLGIEGRGTLEEFSIRDEALLVGTKVTTKAGDKNIENITLEDEVLQFDMNTKDFSYTNPTKTQKVIRDEIYHFEGAGFDQKVSPNHRMIYEQGGEIKECLAKDFEPSEDKYFIIVEGSHMQIKRIKSTDVKITHTKLDEPTEFHCLSVPGKSFVVTDEHGNRSVTGNSMHVEGTGMSDKIIHLTDDSFDTDVLKADGAILVDFWAEWCGPCKMIAPILDEIADEYQGKLTVAKLNIDQNPGTAPKYGIRGIPTLLLFKNGEVAATKVGALSKGQLKEFLDANLA
MBP-wtInt^N-His$_6$ (**1**)	pTT76	pMal-c2x	MBP-SIRDEALLVGTKVTTKAGDKNIENITLEDEVLQFDMNTKDFSYTNPTKTQKVIRDEIYHFEGAGFDQKVSPNHRMIYEQGGEIKECLAKDFEPSEDKYFIIVEGSH$_6$
wtInt^C-eGFP-His$_6$ (**2**)	pSH60	pBAD	MQIKRIKSTDVKITHTKLDEPTEFHCLSVPGKSFVVTDEHGNRSVTGNSMHVEGTVSKGEELFTGVVPILVELDGDVNGHKFSVSGEGEGDATYGKLTLKFICTTGKLPVPWPTLVTTLTYGVQCFSRYPDHMKQHDFFKSAMPEGYVQERTIFFKDDGNYKTRAEVKFEGDTLVNRIELKGIDFKEDGNILGHKLEYNYNSHNVYIMADKQKNGIKVNFKIRHNIEDGSVQLADHYQQNTPIGDGPVLLPDNHYLSTQSALSKDPNEKRDHMVLLEFVTAAGITLGMDELYKGSRSH$_6$
MBP-Int^N-His$_6$ [C81A] (**3**)	pTT92	pMal-c2x	MBP-SIRDEALLVGTKVTTKAGDKNIENITLEDEVLQFDMNTKDFSYTNPTKTQKVIRDEIYHFEGAGFDQKVSPNHRMIYEQGGEIKEALAKDFEPSEDKYFIIVE-H$_6$
MBP-Int^N-His$_6$ [C81S] (**4**)	pTT107	pMal-c2x	MBP-SIRDEALLVGTKVTTKAGDKNIENITLEDEVLQFDMNTKDFSYTNPTKTQKVIRDEIYHFEGAGFDQKVSPNHRMIYEQGGEIKESLAKDFEPSEDKYFIIVEGSH$_6$
Int^C-eGFP-His$_6$ [C124A] (**5**)	pSH102	pBAD	MQIKRIKSTDVKITHTKLDEPTEFHALSVPGKSFVVTDEHGNRSVTGNSMHVE-eGFP-H$_6$
Int^C-eGFP-His$_6$ [C124S] (**6**)	pSH103	pBAD	MQIKRIKSTDVKITHTKLDEPTEFHSLSVPGKSFVVTDEHGNRSVTGNSMHVE-eGFP-H$_6$
Int^C-eGFP-His$_6$ [S+1A] (**7**)	pSH60B	pBAD	MQIKRIKSTDVKITHTKLDEPTEFHCLSVPGKSFVVTDEHGNRSVTGNAMHVE-eGFP-H$_6$
Int^C-eGFP-His$_6$ [N146A;S+1A] (**8**)	pSH60C	pBAD	MQIKRIKSTDVKITHTKLDEPTEFHCLSVPGKSFVVTDEHGNRSVTGAAMHVE-eGFP-H$_6$
Int^C-eGFP-His$_6$ [C124A;N146A] (**9**)	pSH61	pBAD	MQIKRIKSTDVKITHTKLDEPTEFHALSVPGKSFVVTDEHGNRSVTGASMHVE-eGFP-H$_6$
MBP-Int^N-His$_6$ [H68A] (**10**)	pSH139	pMal-c2x	MBP-SIRDEALLVGTKVTTKAGDKNIENITLEDEVLQFDMNTKDFSYTNPTKTQKVIRDEIYHFEGAGFDQKVSPNARMIYEQGGEIKECLAKDFEPSEDKYFIIVE -H$_6$
MBP-Int^N-His$_6$ [S65T] (**11**)	pSH141	pMal-c2x	MBP-SIRDEALLVGTKVTTKAGDKNIENITLEDEVLQFDMNTKDFSYTNPTKTQKVTPNHRMIYEQGGEIKECLAKDFEPSEDKYFIIVE-H$_6$
MBP-Int^N-His$_6$ [S65A] (**12**)	pSH140	pMal-c2x	MBP-SIRDEALLVGTKVTTKAGDKNIENITLEDEVLQFDMNTKDFSYTNPTKTQKVAPNHRMIYEQGGEIKECLAKDFEPSEDKYFIIVE-H$_6$

MBP-Int^N-His_6 [A1G] **(13)**	pSH94	pMal-c2x	MBP-<br>SIRDEGLLVGTKVTTKAGDKNIENITLEDEVLQFDMNTKDFSYTNPTK<br>TQKVIRDEIYHFEGAGFDQKVSPNHRMIYEQGGEIKECLAKDFEPSED<br>KYFIIVE-H_6
Int^C-eGFP-His_6 [T144] **(14)**	pSH138	pBAD	MQIKRIKSTDVKITHTKLDEPTEFHCLSVPGKSFVVTDEHGNRSVAGN<br>SMHVE-eGFP-H_6
MBP-Int^N-His_6 [M70W] **(15)**	pSH96	pMal-c2x	MBP-<br>SIRDEALLVGTKVTTKAGDKNIENITLEDEVLQFDMNTKDFSYTNPTK<br>TQKVIRDEIYHFEGAGFDQKVSPNHRWIYEQGGEIKECLAKDFEPSED<br>KYFIIVE-H_6
MBP-Int^N-His_6 [M70A] **(16)**	pSH106	pMal-c2x	MBP-<br>SIRDEALLVGTKVTTKAGDKNIENITLEDEVLQFDMNTKDFSYTNPTK<br>TQKVIRDEIYHFEGAGFDQKVSPNHRAIYEQGGEIKECLAKDFEPSED<br>KYFIIVE-H_6
MBP-Int^N-His_6 [M70L] **(17)**	pSH118	pMal-c2x	MBP-<br>SIRDEALLVGTKVTTKAGDKNIENITLEDEVLQFDMNTKDFSYTNPTK<br>TQKVIRDEIYHFEGAGFDQKVSPNHRLIYEQGGEIKECLAKDFEPSED<br>KYFIIVE-H_6
Int^C-eGFP-His_6 [G145H] **(18)**	pSH75	pBAD	MQIKRIKSTDVKITHTKLDEPTEFHCLSVPGKSFVVTDEHGNRSVTHN<br>SMHVE-eGFP-H_6
MBP-Int^N-His_6 [R69A] **(19)**	pSH95	pMal-c2x	MBP-<br>SIRDEALLVGTKVTTKAGDKNIENITLEDEVLQFDMNTKDFSYTNPTK<br>TQKVIRDEIYHFEGAGFDQKVSPNHAMIYEQGGEIKECLAKDFEPSED<br>KYFIIVE-H_6
Int^C-eGFP-His_6 [K130A] **(20)**	pSH137	pBAD	MQIKRIKSTDVKITHTKLDEPTEFHCLSVPGASFVVTDEHGNRSVTGN<br>SMHVE-eGFP-H_6
MBP-Int^N-His_6 [E-1A] **(21)**	pSH109	pMal-c2x	MBP-<br>SIRDAALLVGTKVTTKAGDKNIENITLEDEVLQFDMNTKDFSYTNPTK<br>TQKVIRDEIYHFEGAGFDQKVSPNHRMIYEQGGEIKECLAKDFEPSED<br>KYFIIVE-H_6
MBP-Int^N-His_6 [D-2A] **(22)**	pSH113	pMal-c2x	MBP-<br>SIRAEALLVGTKVTTKAGDKNIENITLEDEVLQFDMNTKDFSYTNPTK<br>TQKVIRDEIYHFEGAGFDQKVSPNHRMIYEQGGEIKECLAKDFEPSED<br>KYFIIVE-H_6
Int^C-eGFP-His_6 [M+2A] **(23)**	pSH110	pBAD	MQIKRIKSTDVKITHTKLDEPTEFHCLSVPGKSFVVTDEHGNRSVTGN<br>SAHVE-eGFP-H_6
Int^C-eGFP-His_6 [H+3A] **(24)**	pSH100	pBAD	MQIKRIKSTDVKITHTKLDEPTEFHCLSVPGKSFVVTDEHGNRSVTGN<br>SMAVE-eGFP-H_6
MSIRDE-Int^N-GSH-Int^C-SMHVEGKLGG-Ssp-GyrB[N149A]-CBD	pSH77	pTWIN1	MSIRDEALLVGTKVTTKAGDKNIENITLEDEVLQFDMNTKDFSYTNPT<br>KTQKVIRDEIYHFEGAGFDQKVSPNHRMIYEQGGEIKECLAKDFEPSE<br>DKYFIIVEGSHMQIKRIKSTDVKITHTKLDEPTEFHCLSVPGKSFVVTD<br>EHGNRSVTGNSMHVEGKLGGCFSGDTLVALTDGRSVSFEQLVEEEKQ<br>GKQNFCYTIRHDGSIGVEKIINARKTKTNAKVIKVTLDNGESIICTPD<br>HKFMLRDGSYKCAMDLTLDDSLMPLHRKISTTEDSGHMEAVLNYNH<br>RIVNIEAVSETIDVYDIEVPHTHNFALASTGMKIEEGKLTNPGVSAWQ<br>VNTAYTAGQLVTYNGKTYKCLQPHTSLAGWEPSNVPALWQL

Table S4. Sequence accession codes for class 3 intein proteins from the NCBI nr database of 25 June 2020.

WP_138646874.1	WP_066834705.1	TMF87603.1	WP_112698873.1	BAW10709.1	CRZ17268.1
WP_066834705.1	TMB14016.1	TMA18302.1	WP_112679636.1	APD18035.1	WP_049574304.1
TMB14016.1	TMB36152.1	WP_114016772.1	WP_112675382.1	WP_071621568.1	WP_049560304.1
TMB36152.1	TCN45900.1	WP_101426454.1	WP_107156413.1	APC05445.1	CNE10202.1
TCN45900.1	WP_131944375.1	OGC93480.1	SDD35572.1	SER36982.1	CNF06703.1
WP_131944375.1	RZU89316.1	YP_009619675.1	PIW96417.1	WP_071133870.1	WP_048472480.1
RZU89316.1	WP_067410529.1	WP_097869010.1	TMB08237.1	WP_070732435.1	WP_048341994.1
WP_067410529.1	WP_138199387.1	WP_088644389.1	SCF47535.1	OHE77754.1	YP_009153167.1
WP_138199387.1	WP_132329592.1	WP_097547940.1	TMB25424.1	OHD20030.1	WP_056134538.1
WP_132329592.1	WP_130395882.1	TML77343.1	TMA24068.1	SDQ04363.1	WP_056074179.1
WP_130395882.1	WP_109506974.1	WP_135064576.1	WP_135013931.1	SDS71517.1	WP_056018470.1
WP_109506974.1	RTL17242.1	WP_097232237.1	WP_134038118.1	OGT91356.1	WP_055907072.1
RTL17242.1	WP_121226359.1	WP_133597492.1	WP_132934351.1	OGB70544.1	WP_055906482.1
WP_121226359.1	WP_121008561.1	TCP19786.1	WP_131892270.1	OFX02807.1	WP_055784617.1
WP_121008561.1	WP_118178929.1	WP_135142007.1	WP_113719268.1	OFP74385.1	KRF20595.1
WP_118178929.1	PRD92663.1	RZS91154.1	WP_113689822.1	ODU16637.1	KRE27220.1
PRD92663.1	WP_118611625.1	WP_121157773.1	WP_110240455.1	ODP35065.1	WP_055750737.1
WP_118611625.1	WP_118593641.1	WP_117397983.1	WP_107766358.1	SCK34081.1	KQM04445.1
WP_118593641.1	WP_110103363.1	PYG98806.1	WP_104090845.1	SCK36363.1	WP_055553012.1
WP_110103363.1	RKV97353.1	WP_100350486.1	WP_103941761.1	WP_068609453.1	WP_055547313.1
RKV97353.1	WP_118201512.1	WP_089327701.1	WP_079132544.1	WP_068385443.1	EYT54016.1
WP_118201512.1	QAY14095.1	WP_116896645.1	WP_069990621.1	WP_068190729.1	YP_009012698.1
QAY14095.1	WP_117129550.1	PZO75091.1	TMM30846.1	WP_067963309.1	WP_005273125.1
WP_117129550.1	TAM50267.1	WP_095063836.1	TML25755.1	WP_067819980.1	WP_005147590.1
TAM50267.1	RZO92767.1	WP_065352661.1	WP_135139241.1	WP_067579862.1	WP_005144869.1
RZO92767.1	WP_116136355.1	BBG72166.1	WP_135130129.1	WP_067579158.1	WP_005142194.1
WP_116136355.1	WP_122382386.1	WP_112314092.1	WP_133828212.1	WP_067486580.1	WP_004996404.1
WP_122382386.1	WP_122140994.1	WP_108469412.1	WP_128802974.1	WP_067391432.1	WP_003919772.1
WP_122140994.1	WP_114691659.1	OZA11311.1	WP_123989081.1	WP_067359717.1	WP_003606697.1
WP_114691659.1	WP_068322198.1	OHC10437.1	WP_117378424.1	WP_067307957.1	EOM76929.1
WP_068322198.1	WP_113984957.1	OYW81138.1	WP_107016328.1	WP_067244385.1	WP_001748043.1
WP_113984957.1	WP_132838131.1	WP_071464757.1	WP_106365115.1	WP_067071507.1	GAC80350.1
WP_132838131.1	WP_121003235.1	RKV65836.1	WP_102907409.1	WP_067069574.1	EME15424.1
WP_121003235.1	WP_111667651.1	WP_094181458.1	WP_097320251.1	WP_067041206.1	EMD27977.1
WP_111667651.1	WP_111331798.1	WP_091619288.1	SNX94564.1	WP_067027000.1	YP_007237282.1
WP_111331798.1	WP_071265103.1	WP_106281703.1	OJV84193.1	SCG88010.1	YP_007007266.1
WP_071265103.1	PYN47016.1	WP_089405479.1	WP_068156924.1	OCF88591.1	YP_007001273.1
PYN47016.1	TMB08201.1	WP_075013949.1	WP_123669666.1	WP_064421365.1	YP_006989360.1
TMB08201.1	PYN28641.1	WP_123928459.1	GDL22470.1	WP_064420567.1	YP_007001261.1
PYN28641.1	PYN05502.1	WP_092195799.1	WP_134807586.1	WP_064417779.1	YP_006988682.1
PYN05502.1	PYM84669.1	WP_029373145.1	WP_097753625.1	WP_064413491.1	CCH28817.1
PYM84669.1	PYN35890.1	WP_124265792.1	WP_113975235.1	WP_037370101.1	GAV40833.1
PYN35890.1	PYM81983.1	WP_121186634.1	WP_138636751.1	WP_037175026.1	OLE61828.1
PYM81983.1	PID29378.1	SEO58475.1	TMK50290.1	WP_037137090.1	OLE25249.1
PID29378.1	RUP37414.1	SDP91096.1	TMB10056.1	WP_036964283.1	WP_074329300.1
RUP37414.1	PYN67039.1	WP_132404500.1	WP_132205644.1	WP_036955242.1	WP_074303990.1
PYN67039.1	OGK87146.1	WP_131737306.1	WP_121791788.1	WP_036554298.1	WP_073906012.1
OGK87146.1	RAS68147.1	WP_130780064.1	WP_114201979.1	WP_036496203.1	WP_073905700.1
RAS68147.1	WP_118264606.1	WP_124344070.1	WP_106244137.1	WP_036482937.1	WP_073889737.1
WP_118264606.1	WP_117959186.1	WP_116247523.1	WP_103561322.1	WP_036342191.1	WP_073877082.1
WP_117959186.1	WP_117600650.1	WP_113705041.1	PIY08587.1	WP_035947340.1	WP_073867910.1
WP_117600650.1	PTX39093.1	WP_101787566.1	ASV44090.1	WP_035936258.1	WP_073831840.1
PTX39093.1	QBI99281.1	SDL53865.1	WP_089311086.1	WP_035917940.1	WP_073801431.1
QBI99281.1	QAY03708.1	WP_067793489.1	WP_075024298.1	WP_035912994.1	WP_073773416.1
WP_138646874.1	YP_009286689.1	WP_137782078.1	WP_067367782.1	WP_035776153.1	WP_073760822.1
WP_066834705.1	PPB79369.1	TAL21641.1	WP_066929568.1	WP_035718223.1	WP_073735969.1
SCE70414.1	AUR97927.1	WP_132339176.1	WP_132512779.1	WP_034716904.1	WP_073724517.1
WP_137802620.1	WP_125640613.1	WP_131980854.1	WP_132340455.1	WP_034221330.1	WP_073464085.1
WP_135837781.1	WP_121517900.1	WP_131304392.1	WP_132268477.1	WP_033436337.1	WP_073381317.1
TDW71489.1	RAJ66498.1	WP_130461048.1	WP_132184365.1	WP_033428224.1	WP_073216906.1
WP_131363537.1	WP_107073925.1	RZT87609.1	WP_132178712.1	WP_033290508.1	WP_073105606.1
QBI56714.1	WP_105550856.1	RYM27481.1	WP_132150133.1	WP_033246573.1	WP_072920539.1
RZT14637.1	WP_090587372.1	WP_129309253.1	WP_132048711.1	KGI70518.1	WP_063798466.1
WP_129186887.1	WP_088643681.1	WP_128428406.1	WP_131980703.1	KGI68914.1	ANC71580.1
AYY15761.1	WP_078895593.1	WP_128136492.1	WP_131965828.1	GAK37465.1	WP_063482012.1
WP_122192322.1	WP_063765198.1	WP_126712864.1	WP_131954576.1	WP_029949587.1	WP_081609479.1
WP_111250926.1	WP_030452638.1	WP_125934781.1	WP_131877621.1	WP_029893345.1	WP_081607597.1
WP_106184954.1	WP_133851634.1	WP_125056339.1	WP_131754947.1	WP_029478013.1	WP_081596357.1
WP_089948390.1	WP_129911214.1	WP_124442391.1	WP_131307802.1	WP_028653754.1	WP_081552286.1
WP_070190344.1	WP_089950741.1	WP_123743569.1	WP_130631241.1	WP_028632697.1	WP_081494012.1
WP_029253835.1	WP_067915002.1	WP_123495814.1	WP_130405778.1	WP_028474367.1	OQS19478.1
WP_137986840.1	CCH78756.1	WP_123240531.1	WP_130328525.1	WP_028433410.1	WP_081341673.1
WP_135996682.1	CCI53590.1	WP_120023328.1	WP_130011802.1	WP_028432545.1	WP_081314608.1

TEX50685.1	TDV54133.1	WP_119663725.1	WP_129426823.1	WP_028051077.1	WP_081033088.1
RWX45674.1	WP_132755318.1	WP_119314614.1	WP_129387456.1	WP_028045847.1	WP_081026081.1
RKZ99057.1	WP_131950698.1	WP_117229502.1	WP_128222016.1	WP_027773470.1	WP_080678510.1
PZU44595.1	WP_128838613.1	RDV52152.1	WP_127357548.1	WP_027752912.1	ARB15565.1
WP_097517357.1	WP_127465537.1	WP_111182217.1	WP_125612766.1	WP_027749010.1	WP_080041941.1
WP_097456137.1	WP_125659240.1	WP_107423138.1	WP_125037112.1	WP_043605099.1	OPY74932.1
WP_094805220.1	WP_125619821.1	WP_107155709.1	WP_123915761.1	WP_043201382.1	WP_079676369.1
WP_128979849.1	WP_124706574.1	WP_107136746.1	WP_123816131.1	KIR64373.1	WP_079609152.1
WP_125819475.1	WP_116211726.1	WP_106849239.1	RPE33627.1	WP_041561896.1	WP_079574839.1
WP_123562433.1	WP_116205972.1	WP_103953953.1	WP_123553919.1	WP_040865634.1	WP_079574046.1
WP_118945527.1	REH42610.1	GBF04943.1	ROS26263.1	WP_040862456.1	WP_079411909.1
WP_100566922.1	SUE30098.1	WP_101367541.1	WP_123110211.1	WP_040839197.1	WP_079251991.1
ORB05940.1	WP_114057502.1	WP_100560572.1	WP_122149629.1	WP_040829179.1	WP_079171714.1
WP_069953784.1	WP_112453494.1	PIG54497.1	WP_121889502.1	WP_040711511.1	KZP88195.1
WP_069398904.1	WP_112229095.1	WP_093407509.1	WP_121192091.1	WP_040541809.1	BAU83147.1
WP_067111917.1	WP_109778822.1	WP_091664886.1	WP_120695724.1	WP_040519030.1	SAK58981.1
WP_066815746.1	WP_109198403.1	WP_091442369.1	WP_120690092.1	WP_040514332.1	WP_063042551.1
WP_136205753.1	WP_108637964.1	WP_091274920.1	WP_120061745.1	KIH98766.1	WP_083562693.1
WP_135358883.1	WP_106675084.1	WP_089336816.1	WP_119927588.1	WP_039823488.1	WP_083449856.1
TFW66612.1	WP_106192621.1	WP_081851616.1	RIW42221.1	WP_039380679.1	WP_083442284.1
WP_132340605.1	WP_104991932.1	SDG96052.1	RGC68677.1	KID28621.1	WP_083267962.1
WP_120754517.1	PPT13102.1	OFW79211.1	WP_115941513.1	WP_037978237.1	WP_083178505.1
WP_120568507.1	WP_103533600.1	SCK39689.1	REF29395.1	WP_037975639.1	WP_083164286.1
WP_116507954.1	WP_102916594.1	SCG78263.1	REE97228.1	WP_037928277.1	WP_083159699.1
WP_102143887.1	WP_101783405.1	WP_065119461.1	WP_113838388.1	WP_037785472.1	WP_083157492.1
WP_101424582.1	ATZ26759.1	WP_065070428.1	WP_113835324.1	WP_037717039.1	WP_083147131.1
WP_101412358.1	WP_099282058.1	WP_065036922.1	WP_112724971.1	WP_037625041.1	WP_083132244.1
WP_097187743.1	WP_099023840.1	WP_064930888.1	WP_111254803.1	EDX22563.1	WP_083126486.1
WP_095579870.1	WP_098514132.1	SBT64935.1	WP_111176115.1	YP_001648921.1	WP_083125297.1
WP_091300823.1	WP_098026515.1	WP_030601798.1	PZV95100.1	EAX47548.1	WP_083117755.1
WP_091104078.1	WP_097956606.1	TMS00410.1	WP_110909083.1	YP_418073.1	WP_083109782.1
WP_091048878.1	WP_096491903.1	TML22694.1	PYN94417.1	YP_418072.1	WP_083095156.1
WP_079123138.1	WP_090006119.1	TLS55349.1	WP_110207217.1	Q9F5P4.1	WP_083086903.1
SDY93818.1	SEL74357.1	WP_137451473.1	WP_110050868.1	WP_018654196.1	WP_083071535.1
WP_068159740.1	WP_067478127.1	WP_136446384.1	WP_109805280.1	WP_018599306.1	WP_083062143.1
WP_065149930.1	WP_067381192.1	WP_135132047.1	WP_109282303.1	WP_018330395.1	WP_083048218.1
WP_065130638.1	SCF72900.1	WP_132631392.1	WP_109132444.1	WP_018178867.1	WP_083010400.1
WP_064874909.1	WP_065913777.1	WP_132597091.1	WP_107701209.1	WP_018157578.1	WP_082993967.1
WP_136563682.1	KOG66222.1	RLA58348.1	WP_128801833.1	WP_018111625.1	WP_064395156.1
WP_107262515.1	WP_138671462.1	PZV05771.1	WP_125491694.1	WP_018103258.1	WP_064314987.1
WP_106966321.1	TMA76939.1	ODS97740.1	WP_123991841.1	WP_017869889.1	WP_063987619.1
WP_106584813.1	WP_138206263.1	WP_131122032.1	ROQ13732.1	WP_017794060.1	WP_063935522.1
WP_106538558.1	WP_137161862.1	WP_130299227.1	WP_119099576.1	WP_017614214.1	WP_063854576.1
WP_106438286.1	WP_133872622.1	WP_112472261.1	WP_116158647.1	WP_017609392.1	WP_061290581.1
PRY10793.1	WP_067451495.1	WP_111603335.1	WP_110631051.1	WP_017587494.1	WP_061263509.1
WP_103957122.1	WP_066935601.1	WP_107441737.1	WP_103548554.1	WP_017577051.1	WP_061006636.1
WP_102509791.1	WP_066583366.1	SDG46361.1	WP_099935868.1	WP_017546298.1	WP_061003417.1
WP_101833872.1	SCF36556.1	WP_070010739.1	WP_093593219.1	WP_017539580.1	KXB91592.1
WP_101396228.1	KOG89057.1	WP_030802383.1	WP_071370690.1	WP_016906129.1	YP_009222325.1
WP_101187521.1	WP_030880876.1	WP_030414210.1	WP_067918463.1	WP_016435320.1	WP_060643502.1
PKK11838.1	WP_030445241.1	WP_030381572.1	WP_065916637.1	WP_015750427.1	WP_060353149.1
PKA92460.1	WP_029340299.1	WP_128435156.1	WP_031485162.1	WP_015619657.1	WP_059421936.1
WP_100498011.1	WP_028984618.1	WP_123455582.1	WP_030851987.1	WP_015421570.1	WP_059233969.1
WP_099848447.1	WP_089927502.1	WP_115905666.1	WP_030773520.1	WP_015328402.1	WP_059203095.1
WP_098752902.1	RYT90325.1	RDI32852.1	WP_030611600.1	WP_015307921.1	WP_059165468.1
WP_098747558.1	WP_118306985.1	WP_107459323.1	WP_030018376.1	WP_015104513.1	EJF46495.1
WP_098455704.1	TME84322.1	WP_104781445.1	WP_130468552.1	WP_015051376.1	CCF97565.1
WP_097634371.1	WP_127275200.1	WP_102913975.1	WP_129906966.1	WP_014798202.1	GAB08270.1
WP_097207520.1	WP_108127850.1	PKW43869.1	WP_120280874.1	CTQ93867.1	EHI39167.1
WP_097182910.1	WP_107137742.1	WP_097220969.1	AXY54765.1	WP_052865184.1	EGZ49193.1
WP_094460331.1	RKZ06952.1	SOE30748.1	AYA24452.1	WP_052859923.1	CCA85592.1
WP_094449366.1	WP_102127182.1	WP_094055746.1	RDI18900.1	WP_052747585.1	BAJ29682.1
WP_093711986.1	WP_126242451.1	WP_090008439.1	WP_114451277.1	WP_052624859.1	ADL48355.1
WP_093657175.1	WP_123036742.1	WP_089312898.1	WP_111832721.1	WP_052457553.1	ADB35972.1
WP_093583048.1	WP_115537095.1	KJK37754.1	WP_102800532.1	WP_052445547.1	EEP72513.1
WP_093409379.1	WP_102608215.1	WP_030298502.1	WP_101368795.1	WP_052424324.1	BAH51468.1
WP_093379403.1	ODU62205.1	WP_030206282.1	WP_099020864.1	WP_052103304.1	WP_014743487.1
WP_093180832.1	WP_094551784.1	WP_028814584.1	WP_095567418.1	WP_051778050.1	WP_049925101.1
WP_092624348.1	WP_099747961.1	WP_103090948.1	WP_094601397.1	WP_051764375.1	WP_049915575.1
WP_092198755.1	WP_088249746.1	RCT00012.1	WP_092977133.1	WP_051711805.1	AKS31583.1
WP_091945968.1	OZV79318.1	WP_101574963.1	WP_092928742.1	WP_051621782.1	WP_049715776.1
WP_091930851.1	WP_091044103.1	WP_113780765.1	WP_090006593.1	WP_051515098.1	OKH84318.1
WP_091667474.1	WP_133304554.1	WP_092602718.1	WP_067808441.1	WP_051411228.1	OKH81135.1
WP_091541938.1	WP_091070991.1	WP_133412691.1	WP_067628232.1	WP_051306506.1	OJV59237.1
WP_091432385.1	WP_130345138.1	WP_116916214.1	WP_028928659.1	WP_051244015.1	OJV24762.1
WP_091377878.1	WP_120081766.1	PZU03109.1	WP_138251323.1	WP_051105087.1	APH03252.1

WP_091199685.1	WP_106115166.1	WP_132293392.1	WP_138232365.1	WP_051053550.1	WP_071948376.1
WP_091160635.1	WP_095785663.1	TCO27775.1	TLG18080.1	WP_050749683.1	WP_071930953.1
WP_091127540.1	STZ46562.1	WP_130461288.1	WP_137256717.1	WP_050545156.1	SHN84688.1
WP_091112928.1	WP_104864197.1	WP_129975144.1	WP_137146427.1	WP_050066718.1	WP_027741611.1
WP_091044141.1	ATA27194.1	WP_123303452.1	WP_136340912.1	WP_013473275.1	WP_027506023.1
WP_090937721.1	WP_093350917.1	WP_109472765.1	WP_135127533.1	WP_013171746.1	WP_026923972.1
WP_089154845.1	WP_067894335.1	PTU56559.1	WP_133979965.1	WP_013118883.1	WP_026918140.1
WP_089004749.1	WP_065370962.1	WP_107268409.1	TDW89548.1	WP_012951013.1	WP_026820363.1
WP_089002827.1	SCI96774.1	WP_104431614.1	TDW80900.1	WP_012836326.1	WP_026535902.1
WP_088996662.1	WP_074662691.1	WP_083371400.1	TDW18027.1	WP_012620017.1	WP_026412370.1
WP_088987068.1	WP_122327325.1	WP_072740460.1	TDU06897.1	WP_011891781.1	WP_026385612.1
OJV83172.1	WP_092265002.1	SCG73352.1	TDO67470.1	WP_011607882.1	KDS72398.1
SFL63351.1	WP_119447129.1	WP_135557871.1	TDO54775.1	WP_011562736.1	WP_026248379.1
SEN07263.1	OYQ15431.1	WP_134915806.1	WP_132414480.1	WP_011483945.1	WP_026125279.1
SDH72343.1	SEG12596.1	WP_108961703.1	WP_132326509.1	WP_010849428.1	WP_026122436.1
SDS74332.1	TME09835.1	PZO57332.1	WP_132165597.1	WP_010695433.1	WP_026116317.1
SCL60627.1	TAM77882.1	AXQ68523.1	WP_131513042.1	WP_010539522.1	WP_026114426.1
WP_067894208.1	PYG42745.1	WP_136723155.1	WP_131285013.1	WP_010312872.1	WP_025738219.1
WP_134885479.1	PVZ11133.1	WP_136231825.1	TCO46313.1	WP_009679798.1	KDN76300.1
QBB06080.1	SDN98278.1	GDY62505.1	TCO24615.1	WP_009655478.1	WP_024801532.1
WP_117581937.1	SCE75136.1	WP_134036407.1	WP_131106278.1	WP_009441951.1	WP_024755910.1
WP_117032795.1	WP_091294149.1	WP_132154217.1	RZU20470.1	WP_009438459.1	WP_024491632.1
WP_112018343.1	VED60408.1	TCU32208.1	WP_125079500.1	WP_008835326.1	WP_024444218.1
WP_088959702.1	WP_117734088.1	TCR18813.1	WP_123027514.1	WP_007617542.1	AQT82409.1
RMF63626.1	WP_068948004.1	WP_128977477.1	WP_120739217.1	WP_007453812.1	WP_077860589.1
GCA97377.1	WP_112276251.1	WP_040127541.1	WP_065470963.1	WP_007269386.1	WP_077854459.1
WP_117356418.1	AWH96944.1	WP_137722955.1	WP_064943458.1	WP_006696216.1	WP_077692621.1
WP_114739452.1	WP_107757795.1	WP_135358698.1	WP_064932227.1	WP_006377163.1	WP_077689354.1
WP_112684936.1	PIB73353.1	TDP66083.1	WP_064629135.1	WP_006332631.1	AQS69681.1
WP_112255106.1	WP_099252288.1	TDP43243.1	KMS86608.1	WP_006330443.1	WP_077384753.1
WP_112245484.1	WP_099041561.1	WP_132992569.1	WP_029930205.1	WP_006306642.1	OOL30423.1
RBO91547.1	WP_095786164.1	WP_132672015.1	WP_105327472.1	WP_006242249.1	OOK76376.1
WP_110037833.1	WP_095719329.1	WP_131734509.1	WP_090045788.1	WP_005944428.1	OOK71065.1
SPM29741.1	WP_093595227.1	WP_128145013.1	QCR82057.1	WP_014378669.1	WP_077033333.1
WP_106265822.1	WP_088304327.1	WP_127917758.1	WP_136170642.1	WP_014362023.1	WP_076993121.1
PRY52526.1	SEB17350.1	WP_125727256.1	WP_135234058.1	WP_059159053.1	WP_076991101.1
WP_101952866.1	WP_069393905.1	WP_125687080.1	WP_134047751.1	WP_059093923.1	ONI67890.1
PLC13508.1	WP_068111882.1	WP_125679841.1	WP_130918293.1	WP_059092397.1	WP_076479229.1
WP_101465153.1	OBG15221.1	RRQ28484.1	WP_128138429.1	WP_059035027.1	WP_076468431.1
SNY87447.1	OBF38084.1	ROP39378.1	WP_124714056.1	KUH96291.1	WP_076404168.1
WP_095764237.1	WP_030465681.1	RKT84572.1	WP_122494196.1	YP_009215212.1	WP_076216599.1
WP_094289502.1	WP_029112246.1	WP_120040646.1	WP_120328345.1	WP_058857601.1	WP_076091262.1
WP_092862369.1	WP_137872359.1	WP_116707955.1	WP_119583953.1	WP_058856134.1	WP_076020580.1
ORB04150.1	WP_132161594.1	WP_116043085.1	WP_117406524.1	KUG57062.1	WP_075959204.1
SFS86334.1	RZL80221.1	WP_115966612.1	WP_116510978.1	GAQ74039.1	WP_075952559.1
WP_071286433.1	WP_127962094.1	REH49822.1	WP_110564814.1	GAQ22572.1	WP_075921343.1
WP_070945663.1	WP_124709162.1	REG77641.1	WP_110314116.1	KSU56595.1	WP_075920277.1
WP_070184906.1	WP_124389603.1	AXK77503.1	SPM39475.1	WP_057615821.1	WP_075849845.1
WP_069414354.1	WP_123927197.1	RDI63073.1	WP_104360332.1	WP_057580495.1	WP_075829641.1
WP_068293580.1	WP_121872805.1	RDI30465.1	WP_102805857.1	WP_057478314.1	WP_075735248.1
WP_068236179.1	WP_121118550.1	WP_112279660.1	ASW88513.1	WP_057443746.1	OLF04305.1
WP_068001760.1	WP_115317918.1	WP_110472300.1	WP_094483923.1	WP_056823935.1	WP_082658595.1
WP_067776380.1	WP_114739635.1	WP_109237986.1	WP_092626636.1	WP_056808806.1	ORC25089.1
WP_067439986.1	WP_112555878.1	WP_106958491.1	WP_091041833.1	WP_056765071.1	WP_082647624.1
WP_067373949.1	WP_111510553.1	WP_106955269.1	WP_090070492.1	WP_056680434.1	ORA75495.1
WP_067235034.1	PZS32955.1	WP_105386979.1	ODT98357.1	WP_056586468.1	ORA56650.1
WP_066977365.1	SQI34073.1	WP_104391995.1	WP_069401079.1	WP_056551390.1	ORA11582.1
WP_066843309.1	WP_108057909.1	WP_102419218.1	WP_068057361.1	EXI92823.1	WP_082614363.1
WP_065155925.1	WP_105940843.1	SOX56819.1	WP_068018978.1	GAF50369.1	WP_082600503.1
WP_065151464.1	WP_105420082.1	WP_099949561.1	WP_067820867.1	CCH74255.1	OQZ05627.1
WP_065145431.1	WP_102144334.1	WP_098003740.1	WP_067415018.1	ETZ45592.1	WP_082221095.1
WP_065140243.1	WP_101819901.1	PBI95566.1	WP_067271722.1	WP_023549667.1	OQY43791.1
WP_065130471.1	WP_098959605.1	WP_094676930.1	WP_067007433.1	WP_023529438.1	WP_082176762.1
WP_065041924.1	WP_096312600.1	WP_094644115.1	WP_066851804.1	EST26039.1	WP_082154942.1
WP_065034253.1	WP_094647574.1	WP_094634942.1	WP_065029505.1	WP_023294191.1	WP_082131745.1
WP_065015619.1	WP_094608886.1	WP_094621830.1	WP_065023235.1	WP_022920106.1	WP_082011108.1
WP_064945910.1	WP_092804981.1	WP_094611445.1	WP_031936839.1	WP_021608202.1	WP_082010187.1
WP_064854054.1	WP_092525476.1	WP_094024664.1	WP_064491230.1	WP_021604939.1	WP_082004216.1
WP_030197837.1	SKT94852.1	WP_093264307.1	WP_102492983.1	EPX84024.1	WP_081993606.1
WP_131312044.1	WP_070947784.1	WP_089299422.1	WP_133755422.1	WP_020645735.1	WP_081978956.1
WP_131207971.1	SEC89332.1	ORC22384.1	QAY16501.1	WP_020469620.1	WP_081970837.1
WP_117768683.1	AOW94028.1	SFA50978.1	WP_127837222.1	WP_020161272.1	WP_081883820.1
KFI63676.1	WP_070355773.1	SED82587.1	AYB70223.1	WP_020135058.1	WP_081883411.1
TDH54705.1	ODV09115.1	WP_068919362.1	STY77486.1	WP_020121001.1	WP_081879332.1
WP_132063503.1	WP_065493345.1	WP_068147297.1	PZU87338.1	WP_019886178.1	WP_081861462.1
TCW21879.1	WP_064868762.1	WP_068119892.1	WP_101429844.1	WP_019856357.1	WP_081714530.1

TCO64724.1	WP_124861781.1	WP_067826307.1	WP_092486895.1	WP_019634813.1	WP_081706086.1
WP_126336504.1	RRA14763.1	WP_067720449.1	PZN07242.1	WP_019631638.1	WP_081665040.1
WP_125092476.1	WP_124583674.1	WP_067567659.1	WP_138687628.1	WP_019550113.1	SMD15951.1
WP_116539117.1	WP_124477296.1	WP_067532849.1	WP_133798326.1	WP_019548092.1	WP_084501549.1
WP_116373020.1	WP_071334602.1	WP_067256315.1	WP_130291625.1	WP_019356724.1	WP_084498820.1
SUA31367.1	WP_069229679.1	WP_067131387.1	QAY14121.1	WP_019068738.1	WP_084495899.1
QAY12509.1	WP_130416071.1	PZM98728.1	WP_087909121.1	WP_019011006.1	WP_084488359.1
RTL03100.1	RZU33770.1	AWV48884.1	WP_087702218.1	WP_018838791.1	WP_084483081.1
AYR01443.1	RWU84101.1	WP_110746306.1	OUU23256.1	WP_018714108.1	WP_084457883.1
AYQ99944.1	WP_126808593.1	WP_110647583.1	WP_087555968.1	WP_018713764.1	WP_084412834.1
RLI97986.1	RUR74919.1	PYN76458.1	WP_087470404.1	WP_014210196.1	WP_084377127.1
AXF51495.1	RUL91593.1	PYM46678.1	WP_087076248.1	WP_014137000.1	WP_084345819.1
AWH13822.1	RTL63016.1	PYM35075.1	ART67263.1	AKN15637.1	WP_084343295.1
WP_070122565.1	RTK97721.1	PXX70288.1	WP_086818672.1	WP_047893530.1	WP_084282272.1
WP_109523679.1	VEI90740.1	PXX65562.1	WP_086801806.1	WP_047868367.1	WP_084259300.1
QBQ74860.1	VEG58677.1	WP_109656957.1	WP_086756045.1	AKL66081.1	WP_084223094.1
WP_101404706.1	VEG42012.1	WP_109058030.1	WP_086719369.1	WP_047332865.1	WP_084037576.1
WP_100899106.1	VEG31785.1	WP_108999348.1	WP_086667735.1	WP_047015736.1	WP_084033315.1
SEG35269.1	WP_125618489.1	WP_108645700.1	WP_086666976.1	AKH17681.1	WP_084020232.1
TML03254.1	RPI26387.1	PUX09637.1	WP_086599990.1	KKW10136.1	WP_084001987.1
WP_135282738.1	ROP21243.1	WP_108127946.1	WP_086150115.1	KKU05055.1	WP_084000042.1
WP_099065909.1	ROP21242.1	WP_107500573.1	WP_086047938.1	WP_046563722.1	WP_083962283.1
YP_009622170.1	WP_122976782.1	WP_107421366.1	WP_086007602.1	WP_046362148.1	WP_083951678.1
QBZ71922.1	VAZ64356.1	SPL93778.1	WP_086007316.1	WP_046330541.1	WP_083921748.1
RTK94091.1	VBA31668.1	PSO46514.1	WP_086006500.1	CQD03012.1	WP_083913937.1
RMF57584.1	VBA45288.1	WP_105728939.1	WP_086003036.1	WP_046258861.1	WP_083905065.1
RME56231.1	VAZ87833.1	WP_104959203.1	WP_085998960.1	WP_045844005.1	WP_083885477.1
RKZ05678.1	RMH20480.1	PPS42029.1	WP_085998669.1	WP_045824832.1	WP_083864136.1
AYD81189.1	WP_121890810.1	AUR98797.1	WP_085997400.1	GAO06628.1	WP_083837302.1
WP_114119973.1	WP_121829439.1	AUR91592.1	WP_085985687.1	CKI67314.1	WP_083768242.1
WP_110676579.1	WP_121433431.1	WP_101575044.1	WP_085955543.1	CKH13675.1	WP_083741563.1
WP_106138155.1	WP_121181655.1	AUH67564.1	WP_085925195.1	KJK47961.1	WP_062311232.1
WP_102689281.1	RLG45047.1	WP_101401410.1	WP_085564674.1	WP_044362820.1	WP_062144388.1
WP_097052248.1	RLD60460.1	PJE00754.1	WP_085408358.1	WP_044294504.1	WP_062043232.1
WP_138640281.1	RLA58705.1	ATW59327.1	WP_085305824.1	WP_043986349.1	WP_062008022.1
WP_138392497.1	RKZ09778.1	PIQ55384.1	WP_085270451.1	WP_043966791.1	SHU10454.1
TME00821.1	RKX63591.1	WP_099702967.1	WP_085267027.1	WP_043717875.1	SIK65394.1
TMD50565.1	RKV96740.1	PIJ32378.1	WP_085241017.1	WP_055516289.1	SIN38857.1
TMB49443.1	RKV76335.1	PIB80312.1	WP_085233908.1	WP_055509026.1	SBO93500.1
TMA17468.1	RJQ04432.1	PIB78741.1	WP_085222195.1	WP_055154748.1	OPE47476.1
YP_009636920.1	WP_118939886.1	WP_099162049.1	WP_085207307.1	WP_055053121.1	WP_078510932.1
TLH60669.1	WP_118499804.1	PHM69552.1	WP_085199230.1	WP_054678978.1	WP_078495764.1
TLF60656.1	AXV10334.1	PHM52357.1	WP_085184813.1	WP_054371796.1	WP_078126635.1
WP_137107668.1	WP_117616070.1	PFH03690.1	WP_085177034.1	ALG87676.1	WP_082937277.1
YP_009626171.1	WP_117377859.1	ATD72667.1	WP_085161028.1	CUQ37747.1	GAW53601.1
YP_009626071.1	AXQ68989.1	PBA33246.1	WP_085148783.1	WP_054296257.1	WP_082690510.1
YP_009602116.1	WP_116451561.1	ASV44554.1	WP_085128972.1	WP_054289516.1	WP_082681234.1
BBJ50476.1	AXO21210.1	ASU81717.1	WP_085110066.1	WP_054260532.1	WP_084542310.1
WP_135282593.1	AXN53138.1	ASU80481.1	WP_085081859.1	WP_054053208.1	WP_084524994.1
TGA74150.1	AXK35324.1	OZE20364.1	WP_085080108.1	WP_053928565.1	WP_084512670.1
TFH11188.1	WP_114906766.1	OZC77117.1	WP_085079988.1	WP_053777617.1	WP_084505254.1
WP_134256215.1	SUB10447.1	OZC31070.1	WP_085074399.1	KOX90918.1	WP_062430519.1
TDU35813.1	STZ34542.1	SNW20809.1	WP_085072649.1	WP_053747972.1	WP_062394615.1
TDN77537.1	SUA03321.1	WP_092563841.1	OSC73643.1	WP_053746283.1	WP_072583118.1
QBP07356.1	STZ59640.1	WP_092351403.1	WP_084965046.1	WP_053737792.1	WP_072487258.1
WP_133279304.1	AXG14891.1	WP_091454000.1	WP_084955414.1	WP_053723102.1	WP_078893910.1
TDH17561.1	AXC34374.1	WP_091088269.1	WP_084892108.1	WP_053722762.1	WP_078761996.1
WP_132573196.1	SRX79173.1	WP_090419400.1	ORW25643.1	WP_053714492.1	WP_082968944.1
WP_132457878.1	RAV04322.1	WP_089802871.1	ORW11126.1	WP_053669169.1	WP_082964595.1
WP_132165424.1	SQA10388.1	WP_089248617.1	ORW09546.1	WP_053614779.1	WP_082951514.1
WP_131888796.1	SPX91644.1	WP_089098526.1	ORV96054.1	WP_053204009.1	WP_084614000.1
VFS60134.1	SPX94387.1	WP_088945973.1	ORV72589.1	WP_053200831.1	WP_084581773.1
WP_131578095.1	RAJ47399.1	WP_088896957.1	ORV08014.1	WP_062981681.1	WP_084546582.1
WP_130511202.1	WP_111025248.1	WP_088441837.1	WP_084796067.1	WP_062978912.1	SKK68375.1
WP_130456540.1	PZP22944.1	WP_088246924.1	WP_084753375.1	WP_062827297.1	WP_072690331.1
WP_084745908.1	WP_082993138.1	WP_079166864.1	WP_072914124.1	WP_062715374.1	WP_062497428.1
WP_084685637.1	WP_082977217.1	WP_079127408.1	WP_072815156.1	WP_062709110.1	WP_062649500.1
WP_084678170.1	WP_082975524.1	SKT55714.1	WP_072805027.1		

SUPPORTING REFERENCES

[1] a) G. Volkmann, X. Q. Liu, *FEBS J.* **2011**, *278*, 3431-3446; b) K. Friedel, M. A. Popp, J. C. J. Matern, E. M. Gazdag, I. V. Thiel, G. Volkmann, W. Blankenfeldt, H. D. Mootz, *Chem. Sci.* **2019**, *10*, 239–251.

[2] E. Krissinel, K. Henrick, *Acta Cryst. Sec. D: Biol. Cryst.* **2004**, *60*, 2256-2268.
